# A Lightweight, Elastic, and Thermally Insulating Stealth Foam With High Infrared‐Radar Compatibility

**DOI:** 10.1002/advs.202204165

**Published:** 2022-10-26

**Authors:** Weihua Gu, Samuel Jun Hoong Ong, Yuhong Shen, Wenyi Guo, Yiting Fang, Guangbin Ji, Zhichuan J. Xu

**Affiliations:** ^1^ College of Material Science and Technology Nanjing University of Aeronautics and Astronautics Nanjing 210016 P. R. China; ^2^ School of Materials Sciences and Engineering Nanyang Technological University 50 Nanyang Avenue Singapore 639798 Singapore

**Keywords:** electromagnetic interference (EMI) shielding, infrared‐radar compatibility, microwave absorption, radar cross section, thermal insulation

## Abstract

The development of infrared‐radar compatible materials/devices is challenging because the requirements of material properties between infrared and radar stealth are contradictory. Herein, a composite of poly(3, 4‐ethylenedioxythiophene):polystyrene sulfonate (PEDOT:PSS) coated melamine foam is designed to integrate the advantages of the dual materials and the created heterogeneous interface between them. The as‐designed PEDOT:PSS@melamine composite shows excellent mechanical properties, outstanding thermal insulation, and improved thermal infrared stealth performance. The relevant superb radar stealth performance including the minimum reflection loss value of −57.57 dB, the optimum ultra‐wide bandwidth of 10.52 GHz, and the simulation of radar cross section reduction value of 17.68 dB m^2^, can be achieved. The optimal specific electromagnetic wave absorption performance can reach up as high as 3263.02 dB·cm^3^ g^−1^. The average electromagnetic interference shielding effectiveness value can be 30.80 dB. This study provides an approach for the design of high‐performance stealth materials with infrared‐radar compatibility.

## Graphical abstract

A high‐performance infrared‐radar compatible stealth composite is fabricated. The actual radar cross‐section performance is rationally simulated via smart computer simulation technology.

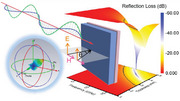

## 1 Introduction

The use of radio frequency (RF) and microwave technology in the form of wireless network and communication systems, radar systems, environmental remote sensing, and medical systems, has resulted in electromagnetic wave pollution.^[^ ^1^, ^2^ ^]^ Radar stealth materials are promising solutions to solve the attendant electromagnetic wave pollution due to their ability to transform electromagnetic energies into heat.^[^ ^3^, ^4^ ^]^ If electromagnetic energies are transformed into heat, the target can be easily detected by infrared detectors.^[^ ^5^ ^]^ Therefore, it is desired to develop materials having both microwave absorption and infrared stealth abilities. However, the requirements of the electromagnetic properties of materials for radar stealth and infrared stealth are mutually restrictive. On the one hand, radar stealth requires materials that strongly absorb electromagnetic waves within a certain frequency range (2–18 GHz), that is, with low reflection and high absorption characteristics.^[^ ^6^ ^]^ On the other hand, infrared stealth requires materials that exhibit high reflection and low absorption characteristics in the infrared band (3–5 µm and 8–14 µm).^[^ ^7^ ^]^ According to Kirchhoff's law,^[^ ^8^ ^]^ infrared emissivity is equal to the absorptivity; and so, infrared stealth requires high reflectivity and low emissivity. These contradictory requirements have created difficulties, especially in the development of broadband microwave absorbing materials that have infrared stealth.

Recently, engineering heterointerfaces have proven to be an effective strategy to design high‐performance infrared‐radar compatible stealth materials.^[^ ^9^, ^10^ ^]^ For instance, Shi et al. have sprayed polyurethane and aluminum powders with low infrared emissivity on a metamaterial substrate with low electromagnetic reflectivity to form an infrared‐radar suppressing coating.^[^ ^11^ ^]^ Huang et al. have fabricated a kind of bionic moth‐eye structural material with multibands adaptability through coating of as‐prepared suspension and follow‐up embossing by honeycomb mold. Its lowest infrared emissivity attains 0.77 and optimal microwave absorption bandwidth ranges from 8.04 to 17.88 GHz (9.84 GHz).^[^ ^12^ ^]^ Zhu et al. have designed a multilayer structural multispectral camouflage device with heterogeneous interfaces, which consists of ZnS/Ge wavelength‐selective emitter and a microwave metasurface.^[^ ^13^ ^]^ Feng et al. proved the feasibility of using multiscale hierarchical metasurfaces to cope with multispectral complementary detection technology by combining theoretical analysis, simulation, and experiment.^[^ ^14^ ^]^ The effectiveness in generating enhanced infrared stealth and appropriate electromagnetic responses can be regulated by tuning the chemical composition and structures. Several mechanisms led by heterointerfaces, such as space charge distribution, electron transport, and lattice defects have been found to have a profound impact on polarization relaxation and conduction loss.^[^ ^15^, ^16^ ^]^ With these advantages, creating hetreointerfaces is a promising approach to improve the infrared‐radar compatible stealth performance. In developing a heterogenous interface of two materials, one used in this work is melamine foam. The porous structure makes the foam intrinsically lightweight and provides a large specific surface area with ample room to load other functional materials.^[^ ^17^ ^]^ The previous work conducted by Jia et al. shows that melamine foams can possess a rough surface that allows for the coating of conductive materials after an alkaline treatment.^[^ ^18^ ^]^ The coating allows a long electromagnetic wave transmission path and enough winding space and generates polarization sites which are instrumental in inducing multiple scattering of microwaves inside the material. In addition, melamine foams are mechanically strong enough as a support material, and they are simple in composition and low in cost.^[^ ^19^ ^]^

The other selected material is an ether substituted conducting polymer poly(3,4‐ethylenedioxythiophene): polystyrene sulfonate (PEDOT:PSS). In general, conducting polymers such as polyaniline, polypyrrole, and polythiophene have pseudo‐metallic behaviors, which are beneficial to low infrared emissivity.^[^ ^20^ ^]^ This can be attributed to Hagen–Rubens law, *E(ω*) = 2(2*ε*_0_ *ω/σ*)^1/2^, where *E(ω)* is infrared emissivity, *ε_0_* signifies permittivity of vacuum, *ω* represents angular frequency, and *σ* stands for electrical conductivity.^[^ ^21^ ^]^ The above conductive polymers are unstable, especially in humid air.^[^ ^22^ ^]^ Among these conductive polymers, ether substituents of PEDOT can reduce the oxidation potential of monomers and polymers, making them easier to polymerize and more stable during redox reactions.^[^ ^23^ ^]^ PEDOT:PSS not only improves the solubility of PEDOT via PSS,^[^ ^24^ ^]^ but also offers a way for tuning the radar stealth and electromagnetic interference (EMI) shielding properties through the optimization of heterogeneous interface. For example, Wang et al. found that PEDOT:PSS‐Fe_3_O_4_‐rGO nanocomposites showed a minimum reflection loss value of ‐61.4 dB and a maximum bandwidth of 6.4 GHz.^[^ ^25^ ^]^ The radar stealth performance of these composites was attributed to the reconstruction of conductive networks for aggregation‐induced charge transport by decorating PEDOT:PSS, and the introduction of heterogeneous interfaces for multiple relaxations. In a similar manner, our team introduced nano‐sulfur particles into graphene‐PEDOT:PSS composites to create a material with multiple interfaces, and obtained a minimum reflection loss value of −21.9 dB.^[^ ^26^ ^]^ In addition, Yu et al. manufactured a series of freestanding PVA/PEDOT:PSS/Ag nanowire films via an evaporation process.^[^ ^27^ ^]^ Due to the high conductivity of both PEDOT:PSS and Ag nanowires, the optimal electromagnetic interference shielding effectiveness (EMI SE) value can reach up to 33 dB. Besides that, Yang et al. fabricated ultralight Ti_3_C_2_T*_x_*/PEDOT:PSS hybrid aerogels with multiple interfaces for high performance EMI shielding, in which EMI shielding was primarily provided by the electromagnetic absorption mechanism.^[^ ^28^ ^]^ These results suggest that PEDOT:PSS is a good option for use in radar stealth and EMI shielding applications.

Given the excellent characteristics of these two materials, it can be deduced that the combination of PEDOT:PSS and 3D structural melamine foam is likely to yield a promising composite material for high‐efficiency infrared‐radar spectrum compatible devices. Herein, effective electrical conductivity introduced by PEDOT:PSS and thermal insulation ability introduced by porous network play dominant roles in infrared stealth mechanisms. The microwave absorption is through sufficient dielectric loss and appropriate impedance matching. Conduction features of the polymers are realized by the linear or planar configuration with conjugated *π* electrons and the role of charge transfer; thereby, adequate dielectric loss can be guaranteed.^[^ ^29^, ^30^ ^]^ With the regulation of loading mass of PEDOT:PSS on melamine foam and the filling of paraffin, the composite shows suitable impedance matching performance.

## 2 Results and Discussion

**Figure 1:**
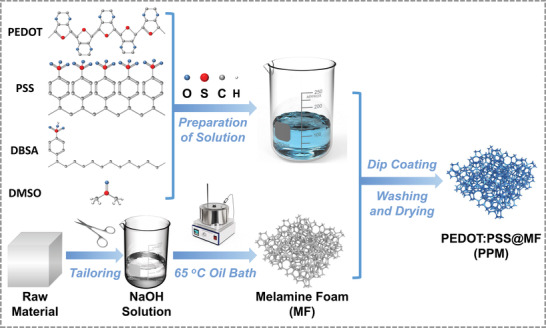
Schematic description of preparing PPM foams.

**Figure** **1** depicts a schematic illustration of the preparation process of the 3D PPM foams. First, the solution used for dip‐coating was made by mixing a pristine PEDOT:PSS suspension, dimethyl sulfoxide (DMSO), and dodecylbenzene sulfonic acid (DBSA). In the final material, PEDOT:PSS plays the role of conductive component, ensuring electrical conductivity for EMI shielding performance and improving dielectric loss for microwave absorption. As PEDOT has hydrophobic features while PSS has hydrophilic features, PEDOT:PSS is not very soluble in most commonly‐used solvents.^[^ ^31^ ^]^ To overcome this issue, DMSO, which is a highly aprotic polar solvent soluble in both water and organic solvents was selected.^[^ ^32^ ^]^ DMSO is known to improve electrical conductivity of this material when used as a cosolvent before being removed by a drying process.^[^ ^33^ ^]^ Besides, DBSA acted as a dispersing agent in this work, which is removed using a washing process.^[^ ^34^ ^]^ At the same time, one whole piece of raw melamine foam (MF) was cut to fit the desired dimensions and cleaned with deionized water and ethanol. After that, the melamine foam was subjected to a hydrophilic treatment using a NaOH solution at 65 °C using an oil bath. The surface of the dried melamine sponges was found to become rougher after treatment, which can be seen from Figure S1, Supporting Information. The final PPM specimens were obtained by dip coating the treated melamine sponges in the PEDOT:PSS solution with the desired dip times, washing process, and drying conditions.

**Figure 2:**
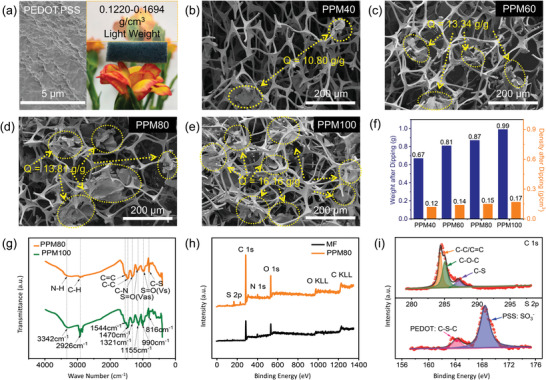
a) SEM image of the pure PEDOT:PSS sample with digital photograph of PPM sample placed on top of an orange flower. b–e) SEM images of PPM40, PPM60, PPM80, and PPM100 foams, respectively. f) Mass and density diagram of the 3D hybrid foams. g) FT‐IR spectra of PPM samples. h) XPS survey scan of pristine melamine sponge and PPM80 specimen. i) High‐resolution of C 1s and S 2p of the as‐prepared PPM80 sample.

Pure PEDOT:PSS samples without melamine foam were also synthesized for comparison. As shown in **Figure** **2**a, the PEDOT:PSS sample exhibits a 2D lamellar structure. With the aid of dispersing agent DBSA and cosolvent DMSO, PEDOT:PSS specimens with enhanced electrical conductivity could be successfully obtained. As illustrated in Figure 2b–e, melamine foams are 3D interconnected networks with nodes formed at the meeting of three/four slender branches. The amount of PEDOT:PSS in the composite increases with dipping time, which can be seen from the SEM images. As depicted in Figure S2, Supporting Information, the surfaces of all PPM samples appear to be fully coated with PEDOT:PSS despite the presence of some aggregation. This can be ascribed to the following reasons: 1) the strong wetting of the hydrophilic MF surface by the as‐prepared PEDOT:PSS solution and 2) the strong intermolecular interactions (*π*–*π* stacking) between MF foam and PEDOT:PSS caused by their aromatic heterocyclic structures.^[^ ^35^ ^]^ In order to fulfill the many design requirements of multifunctional EM absorption and shielding materials, it is necessary to consider physical properties of the material, such as density. In this respect, the synthesized PPM foams perform well, with extremely low densities due to their highly porous nature. Figure 2a demonstrates this by showing the PPM foam supported on top of an orange flower without deforming its petals. As depicted in the column diagram of Figure 2f, the masses of PPM40/60/80/100 specimens are 0.67, 0.81, 0.87, and 0.99 g, respectively. The resulting density values are 0.12, 0.14, 0.15, and 0.17 g cm^−3^ for PPM40, PPM60, PPM80, and PPM100, respectively. The details behind these calculations are presented in Table S1, Supporting Information. The mass loading of PEDOT:PSS on the melamine foams clearly exhibits a gradual increasing trend with increased immersion time, resulting in increased gravimetric loading ratio (*Q*, g g^−1^) (10.80, 13.34, 13.81, and 16.12 g g^−1^ for PPM40/60/80/100).^[^ ^36^ ^]^ The calculations for Q are also presented in Table S1, Supporting Information. As previously mentioned, the lightweight nature of the material may be due to its high porosity. As shown in the mercury intrusion and extrusion curves (Figure S3, Supporting Information) and numerical summary in Table S2, Supporting Information, the void fractions of the PPM foams can reach up to 98.98% (MF), 96.98% (PPM40), 95.84% (PPM60), 95.67% (PPM80), and 92.67% (PPM100), respectively. Figure S3, Supporting Information, also shows that the pore sizes for all samples are mainly distributed within a range of 100 to 200 µm. Pristine melamine foam is found to have an average pore size of 198.003 µm. Meanwhile, the average pore sizes of the PPM40/60/80/100 samples are 137.447, 130.127, 129.840, and 124.443 µm, respectively, indicating decreasing pore size with increasing dipping time. This is likely due to the increased amount of PEDOT:PSS loaded in the composite. That is to say, more filling can create more contact between PEDOT:PSS and the melamine skeleton and air, resulting in decreased porosity and reduced pore size. The FT‐IR transmission spectra of PPM80 and PPM100 porous hybrid foams can be found in Figure 2g. The characteristic peaks at 3342 and 1321 cm^−1^ may be assigned to the stretching vibration of N—H and C—N bonds of pristine melamine foams.^[^ ^37^ ^]^ The peak at around 2926 cm^−1^ corresponds to the C—H chemical bond, which is expected from saturated carbon. Both samples show peaks at 1544, 1470, and 816 cm^−1^, which can be attributed to the C=C, C—C, and C—S chemical bonds of PEDOT. In addition, two peaks centered at 1155 and 990 cm^−1^ may be assigned to the asymmetric and symmetric vibration of S=O functional group, respectively.^[^ ^38^ ^]^ Compared to the peaks of N—H and S=O (Vas) bonds in PPM80, those of PPM100 show a slight shift toward shorter wavelengths (higher wave number). It is well understood that a shift toward higher wave numbers indicates a greater energy required for that bond to vibrate; therefore, suggesting the presence of a more stable functional group. Hence, it can be deduced that PEDOT:PSS can be more firmly loaded on the melamine framework with increased dipping time. XPS was also carried out to provide more information on the chemical states of the synthesized material. The overall XPS spectrum of MF shows C 1s, N 1s, and O 1s photoelectron peaks, and O KLL and C KLL auger peaks, as shown in Figure 2h. Compared with MF, the XPS survey spectrum of PPM80 in Figure 2h presents an additional S 2p peak, which is the result of the presence of PEDOT:PSS. High‐resolution XPS of the C 1s and S 2p peaks from the as‐prepared PPM80 sample are displayed in Figure 2i. Three fitted peaks at 284.6, 286.1, and 287.5 eV are present for C 1s peaks, which may be assigned to C—C/C=C, C—O—C/C—OH, and C—S groups respectively.^[^ ^39^ ^]^ As for the S 2p spectrum, Figure 2i also depicts one peak at ≈164 eV which corresponds to sulfur bonded to carbon (C—S—C) in poly(3, 4‐ethylenedioxythiophen) (PEDOT), while another peak at ≈168 eV corresponds to the sulfur in the SO_3_^−^ group in polystyrene sulphonate (PSS).^[^ ^40^ ^]^ These results show that the as‐prepared lightweight PPM foams not only maintain the 3D porous structure of the MF substrate but also have successfully loaded the PEDOT:PSS conductive polymers.

**Figure 3:**
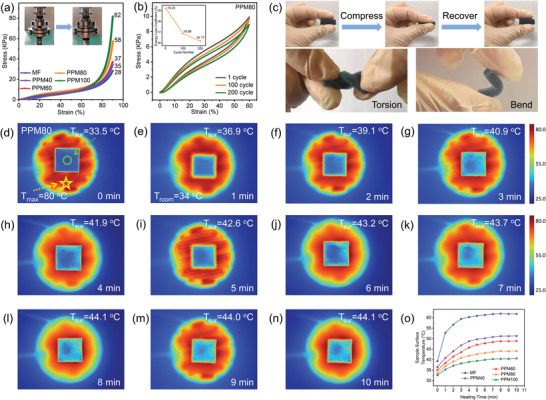
a) Compressive stress–strain (*σ*–*ε*) curves of PPM foams at 90% strain. b) Fatigue test of PPM80 at 60% strain for 200 cycles. Inset: energy loss coefficients over 200 cycles. c) A series of digital images showing compress–recover process and remarkable deformation feature of torsion and bend of the 3D hybrid foams. d–n) Thermal infrared images of PPM80 sample captured at intervals of 1 min from 0 to 10 minutes. o) Variation tendency of the temperature detected on the upper surface of samples versus different heating time at a fixed set temperature of heating platform.

Traditional microwave absorbing coating materials usually show poor durability in harsh environments. In this, due to the 3D cross‐linked network architecture, our lightweight hybrid foam possesses excellent mechanical properties that allows it to withstand repeated uses and effectively recover after deformation. As illustrated in Figure S4, Supporting Information, the PPM80 foam can bear the weight of loads with mass up to 200 g, which is ≈380 times its own (0.5260 g), while only being compressed to 10 mm from the initial 13 mm thickness. The compression resistance of the material was also examined, and the stress–strain (*σ–ε*) curves of PPM hybrid foams are shown in **Figure** **3**a. With a maximum strain *ε* of 90% and an automatic strain rate of 1 mm min^—1^, the maximal *σ* values of MF and PPM40/60/80/100 samples are 28.4, 34.5, 37.3, 57.7, and 82.0 kPa, respectively. Figure S5, Supporting Information; Figure 3b show the compressive mechanical behavior of MF and PPM80 for 200 fatigue cycles with a maximum strain of 60%. After unloading both MF and PPM80, the strain is restored to 0%, that is, the original sample volume is completely restored without plastic deformation. As the number of loading–unloading cycles increases, the energy loss coefficient (dissipated energy of each loop area relative to work done in the loading curve) decreases, which can be seen from the insets of Figure 3b; Figure S5, Supporting Information. The energy loss coefficient values of MF (from 20.4% to 17.15%) are slightly larger than those of PPM80 (from 19.25% to 16.17%), indicating that the addition of PEDOT:PSS positively contributes to the resilience of the composite foam. To further investigate the mechanical properties of the as‐prepared PPM80 specimen, it was subjected to compression, torsion, and bending behaviors by hand, which can be observed in Videos S1–S3, Supporting Information. In addition, Figure 3c contains a series of still frames from the aforementioned videos, revealing the satisfactory ability of the material to recover from compression, and its remarkable ability to undergo torsion and bending. In addition, Figure S6, Supporting Information, depicts the results of a humidity test of three sets of the samples with constant temperature (23 °C) and variable humidity. Upon visual inspection, the three groups of the samples show almost no discoloration, spotting, dissolution or shedding, indicating that the samples are fairly stable under such conditions. There is no doubt that the excellent mechanical properties and respectable humidity resistance of the 3D porous PPM composites will contribute to an excellent service life and expand their fields of application. Besides excellent mechanical performance, the material shows excellent thermal insulation properties, which renders it highly suited for practical applications in high‐temperature conditions. To compare their thermal insulation performance, pristine MF and all hetero‐structural PPM samples were placed on a bulb type heating platform with a set temperature of 80 °C and thermal infrared images were captured from above. The corresponding images after durations of 0 to 10 min are presented in Figure S7, Supporting Information (MF), Figure S8, Supporting Information (PPM40), Figure S9, Supporting Information (PPM60), Figure 3d–n (PPM80), and Figure S10, Supporting Information (PPM100), separately. Among all samples, the pristine MF shows both the greatest surface temperature and the greatest heat transfer rate, which can be seen from the fact that the color of the sample surface quickly turns green, corresponding to increased temperature. As for the PPM hybrid foams, there is a clear progression of decreasing surface temperature as coating time increases, which is evident from Figure 3o. In addition, Figure S11, Supporting Information, shows a thermal infrared image of the side of a PPM80 sample with a thickness of 10 mm. On initially placing this sample on the heating platform, it appeared red on the bottom and blue on the surface. Heating was continued until the surface temperature of the sample was stable. Despite this long heating process, the thickness of the red part at the bottom of the sample remained less than 3 mm, indicating that the sample has good thermal insulation properties. Given the similar external temperature and pressure conditions under which all samples were tested, it is clear that the excellent results of the composites are due to the low thermal conductivity of the PEDOT:PSS component and the open cellular architecture of the MF. The thermal transmission mechanisms of the as‐prepared foams are depicted in Figure S12a, Supporting Information, including thermal conduction in solid phase, thermal conduction between solid phase and gas phase, thermal convection through gas phase, and thermal radiation.^[^ ^17^ ^]^ Figure S12b, Supporting Information, shows that the thermal conductivities of pristine MF, PPM40/60/80/100, and PP are 0.053, 0.036, 0.034, 0.029, 0.025, and 0.020 W m^−1^·K^−1^, respectively, ensuring the possibility of enhanced thermal insulation performance with increasing PEDOT:PSS content. A low density (Table S1, Supporting Information) and high porosity (Table S2, Supporting Information) caused by the 3D network are conducive to the reduction of thermal conduction.^[^ ^41^ ^]^ Furthermore, the abundance of air in randomly distributed pores, which possesses low thermal conductivity, is beneficial to improve thermal insulation by limiting heat transfer to convection and radiation. Videos S4 and S5, Supporting Information, were recorded to further demonstrate the thermal insulation performance of the material. Video S4, Supporting Information, clearly shows that a fresh leaf placed on asbestos wire gauze and heated using an alcohol lamp quickly withered and burned within merely 1 min. However, Video S5, Supporting Information, shows that with the added protection of 3D porous lightweight PPM80 foam, the leaf did not burn even after 10 min but only began to wither after ≈86 s and then turned yellow after 6 min and 19 s. The PPM foams were quite clearly effective thermal insulators. In addition, Table S3, Supporting Information, summarizes the limiting oxygen index (LOI) of all samples and Videos S6–S10, Supporting Information were recorded to show combustion experiments in which all samples were in contact with an open flame. As shown in Table S3, Supporting Information, the LOI values of MF and PPM40/60/80/100 are 33.59%, 31.25%, 30.90%, 26.30%, and 25.19%, respectively. This is consistent with the observations shown in Videos S6–S10, Supporting Information, where MF/PPM40/PPM60 were highly resistant to the flame and PPM80/PPM100 could be ignited but was extinguished immediately after being removed from the fire. This is likely due to the fact that pristine melamine foam is rich in nitrogen atoms, which results in the production of inert gases (N_2_) which serve the purpose of flame retardants when the foam is exposed to open flame. However, PEDOT:PSS also contains some oxygen and sulfur atoms, which may reduce the flame retardant effect. Therefore, it is very important to choose an appropriate proportion of the two components through the dipping time depending on the application's requirements. The infrared stealth properties of these hetero‐structural composites were also studied using thermal infrared images and an infrared emissivity test. On account of the development of middle infrared region (3–5 µm) and thermal infrared band (8–14 µm) detectors, the development of infrared stealth materials capable of overcoming such detectors is of great interest. In order to protect a target from detection, such stealth materials must effectively shield the target's infrared radiation from the detector. In essence, infrared stealth materials must reduce the radiancy difference between the target and the background, Δ*E*, which is simply calculated according to the equation Δ*E* = *E*_t_–*E*_b_,^[^ ^42^ ^]^ where *E*_t_ represents the infrared radiant energy of the target and *E*_b_ signifies the infrared radiant energy of the background. These infrared radiant energies are calculated based on the Stefan–Boltzmann law:^[^ ^43^ ^]^

(1) $$E_{t}=\varepsilon_{t}\sigma T_{t}^{4}$$

(2) $$E_{b}=\varepsilon_{b}\sigma T_{b}^{4}$$

Herein, *ε*_t_ and *ε*_b_ stand for the infrared emissivity of the target and the background, *σ* is the Stefan–Boltzmann constant, and *T*_t_ and *T*_b_ are absolute temperatures of the target and the background. Clearly, radiation energy is in direct proportion to not only infrared emissivity values but also the absolute temperature raised to the fourth power. The performance of our material can be analyzed through its effects on infrared emissivity. It is generally accepted that infrared emissivity is affected by surface roughness, test angle, test temperature, and electrical conductivity.^[^ ^44^–^46^ ^]^ Due to the uniform dipping process, the surface roughness for all PPM samples is likely to be almost the same, while test angle and test temperature are experimentally controlled. Thus, electrical conductivity of the material is likely the key factor informing differences in infrared emissivity. Generally, greater electrical conductivity is associated with reduced infrared emissivity. Table S4, Supporting Information, shows that the resistance values (*R*) of MF and PPM40/60/80/100 specimens are *∞*, 428, 233, 189, and 116 Ω, respectively. Thus, the electrical conductivity values (*σ*) of PPM40/60/80/100 are 0.58, 1.07, 1.32, and 2.16 S cm^−1^, respectively, exhibiting an obvious increasing trend with increased dip‐coating time (Figure S13, Supporting Information). This explains the decreasing trend in infrared emissivity shown in Figure S14a,b, Supporting Information, where the infrared emissivity of pristine MF and PPM40/60/80/100 hybrid foams are 0.872, 0.803, 0.799, 0.793, and 0.788 in the 3–5 µm band and 0.994, 0.823, 0.784, 0.764, and 0.757 in the 8–14 µm band, respectively. Therefore, increased dip‐coating time yields a material with improved infrared camouflage performance. The decreased radiative energy in the above infrared band may lead to accumulated energy inside the material and thermal instability.^[^ ^47^ ^]^ This may cause an increase in the surface temperature of the device, which may render it easily detected by infrared detectors. Therefore, in order to realize infrared stealth, reducing surface temperature of the protected equipment to suppress infrared radiation is another effective method. Thermal imaging was therefore used to demonstrate the PPM foams’ ability to suppress infrared radiation emitted. The thermal infrared images taken of the PPM foams in Figure 3d–n; Figures S7–S10, Supporting Information, show that the surface of the as‐prepared PPM foams has similar color to that of the ambient environment, which is in sharp contrast to the brightness of the heating platform. Clearly, the gas‐filled 3D frameworks of the hybrid foams effectively suppress the visible surface temperature, making them excellent thermal insulators that are well‐suited to infrared camouflage. As a result, it is clear that the 3D lightweight hetero‐structural PEDOT:PSS@melamine composite offers a combination of excellent mechanical properties and good thermal insulation that makes them suited for a wide variety of applications.

The electromagnetic absorption and shielding properties of the PPM foams were also tested. To do this, intact PPM foams were combined with paraffin to form PPM foam/paraffin composites as described in the Experimental Section. According to the transmission line theory, the input impedance (*Z*_in_) represents the impedance value at the air–material interface, which can be calculated by the following formula using the frequency‐dependent complex permeability *μ*_r_ and permittivity *ε*_r_:^[^ ^48^ ^]^

(3) $$Z_{\mathrm{in}}=Z_{M}/Z_{0}={\left(\mu_{r}/\varepsilon_{r}\right)}^{1/2}tanh\left[j\left(2\pi fd/c\right){\left(\mu_{r}\varepsilon_{r}\right)}^{1/2}\right]$$

Above, *Z*_M_ is the characteristic impedance of the microwave absorbing materials, *Z*_0_ is a fixed constant 120 *π* (≈377) Ω standing for the intrinsic impedance of free space, *d* means thickness of the microwave absorber, and *c* signifies the speed of light. Based on the metal back plane model, the microwave‐absorption performance of the bulk PPM/paraffin composites could be evaluated via reflection loss (RL) values:^[^ ^49^, ^50^ ^]^

(4) $$\mathrm{RL}=20\log|\left(Z_{in}-1\right)/\left(Z_{in}+1\right)|$$

**Figure 4:**
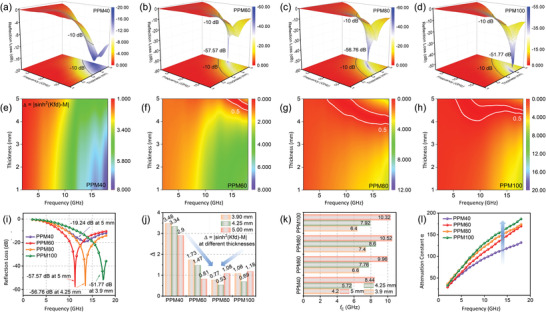
a–d) 3D RL plots of PPM40, PPM60, PPM80, and PPM100/paraffin composites, respectively. e–h) The calculated delta values versus frequency maps (1–5 mm) of a set of PPM/paraffin samples. i) Reflection loss intuitive comparison curves of the 3D hybrid foams. j) Average delta values and k) effective bandwidth comparison column charts of the 3D hybrid foams at the thickness of 3.9, 4.25, and 5 mm. l) Attenuation constant evaluation plots in the frequency range 2–18 GHz.

Composites typically require effective absorption of at least 90% to be described as effective microwave absorbers. In short, the reflection loss values of the composite should be more negative than −10 dB.^[^ ^51^ ^]^ As *Z*_in_ approaches 1, RL values approach −*∞*, which signifies zero reflection and is ideal for the purpose of microwave absorption. As depicted in the 3D RL plots (**Figure** **4**a–d), the PPM40, PPM60, PPM80, and PPM100/paraffin composites all possess satisfactory reflection loss values below −10 dB, which are marked by black bold lines. The minimum RL (RL_min_) values for all PPM/paraffin composites are shown in Figure 4i. In the case of PPM40/paraffin composites, the minimum reflection loss value is only −19.24 dB at 13.4 GHz (in Ku band) for a foam thickness of 5 mm. However, the RL_min_ value of the PPM60/paraffin composites can reach up to −57.57 dB at 11.28 GHz (in X band) for the thickness of 5 mm, which is the minimum RL value among all specimens. As for PPM80/paraffin composites, a RL_min_ value of −56.76 dB can be achieved and the optimal thickness is a comparatively lower at 4.25 mm. In addition, the PPM100/paraffin specimens possess an RL_min_ value of −51.77 dB at 17.36 GHz with an even smaller optimal thickness of 3.9 mm. Although the foam thicknesses are not very thin, this weakness is ameliorated by the fact that the density values of the PPM/paraffin composites are extremely low due to their highly porous 3D interconnected structure as discussed previously. Using Equation (S2), Supporting Information, the specific EM wave absorption performance (SMAP) of the as‐prepared PPM40/60/80/100/paraffin composites could be calculated using the RL values at certain thicknesses within the frequency range 2–18 GHz, and the density of the foam/paraffin composites.^[^ ^52^ ^]^ The density values of the foam/paraffin composites increase with dip coat time as expected from densities of the foam alone, with density values of PPM40/60/80/100/paraffin composites being 0.1220, 0.1377, 0.1471, and 0.1694 g cm^−3^, respectively. As revealed in Table S5, Supporting Information, all samples deliver excellent SMAP values of greater than 2000 dB·cm^3^ g^−1^. In particular, the SMAP value of PPM80/paraffin sample is a very high 3262.02 dB·cm^3^ g^−1^ for a thickness of 3.9 mm revealing its admirable microwave absorption performance. Another metric used in evaluating microwave absorption performance is the degree of impedance matching. It can be calculated using a delta‐function Δ = |sinh^2^(*Kfd*)−*M*|.^[^ ^53^ ^]^ The derivation of this delta‐function is provided through Equations S3–S12, Supporting Information. As *Δ* values of the microwave absorbing materials approach zero, satisfactory impedance matching performance is obtained. The 2D color fill contour *Δ*–*f* curves of intact PPM/paraffin specimens for a thickness range of 1–5 mm are shown in Figure 4e–h, with *Δ* values of 0.5 demarcated by white lines. Apart from PPM40/paraffin composites, all other samples possess at least some portion of low *Δ* values, showing the composites’ high degree of impedance matching at specific thicknesses and frequency ranges. The poor performance of PPM40 can be attributed to an excessively low permittivity caused by the low conductivity of PPM40/paraffin, which prevents electromagnetic waves from traveling inside the material and negatively affects the attenuation and dissipation of electromagnetic waves.^[^ ^54^ ^]^ As shown in Figure 4i, the minimum reflection loss values of each composite can be captured at either 3.9, 4.25 and 5 mm. Therefore, these thicknesses are selected when comparing *Δ* values and effective bandwidth. Column diagrams in Figure 4j further support the conclusion that PPM40/paraffin has poor impedance matching. For these thicknesses, the average values of *Δ* plotted against dipping time form a valley shape. The average *Δ* value of PPM60/paraffin is lower than that of PPM80/paraffin at 5 mm, which may be due to the increased thickness causing reduced electrical resistance and increased microwave reflection from PPM80/paraffin. It can be deduced from this that appropriate thickness contributes to good impedance matching. As previously discussed, electrical conductivity of the composites is also likely to increase with dip time of the PPM foams. Compared with PPM80/paraffin, the higher conductivity of PPM100/paraffin is not in favor of the impedance matching and results in more reflection at the surface of the absorber.^[^ ^55^ ^]^ Due to the role of electrical conductivity in regulating impedance matching (too high or too low are not conducive to impedance matching), it becomes clear that the calculated *Δ* values are consistent with this property, showing an optimal point for PPM80/paraffin at an average *Δ* value of 0.53. Besides that, the engineered interfaces between PEDOT:PSS, 3D porous melamine foam and paraffin may provide long electromagnetic wave transmission paths and sufficient internal reflection space, which facilitates good input impedance. Hence, PPM80/paraffin with its uniformly distributed and moderate amount of conductive component and low paraffin filling shows the appropriate electrical conductivity for the impedance matching at a relatively thin thickness. Effective bandwidth (*f*_E_), which is the range of frequencies for which RL is more negative than −10 dB, is another key metric used to describe microwave absorption performance. In testing this, thickness of samples was restricted to 5 mm in order to avoid excessive mass as would be considered for real‐life applications. As illustrated in Figure 4k, all samples show incrementally increasing bandwidth with increasing thickness. When the thickness is 3.9 mm, all composites possess effective bandwidths larger than 4.2 GHz. If thickness is increased to 5 mm, the specimens display bandwidths greater than 8.44 GHz. Once again, the PPM80/paraffin sample possesses the greatest performance, showing an eye‐catching bandwidth of 10.52 GHz (from 7.48 GHz to 18 GHz), covering the whole X band and Ku band. In this manner, these 3D lightweight PPM/paraffin composites are shown to be excellent candidates for absorbing undesired electromagnetic radiation. The attenuation constant *α* is another key metric for evaluating microwave absorption performance. It can be defined as:^[^ ^56^ ^]^

(5) $$\begin{array}{c} \begin{array}{c} \alpha =\frac{\sqrt{2}\pi f}{c}\times \sqrt{\left(\mu^{\prime \prime }\varepsilon^{\prime \prime }-\mu^{\prime }\varepsilon^{\prime }\right)+\sqrt{{\left(\mu^{\prime \prime }\varepsilon^{\prime \prime }-\mu^{\prime }\varepsilon^{\prime }\right)}^{2}+{\left(\mu^{\prime }\varepsilon^{\prime \prime }+\mu^{\prime \prime }\varepsilon^{\prime }\right)}^{2}}} \end{array} \end{array}$$

As seen from Figure 4l, the average *α* values for the PPM40, PPM60, PPM80, and PPM100/paraffin composites are 90.17, 106.41, 116.22, and 121.92, respectively. Clearly, the *α* values of the composites are proportional to their electrical conductivity. The excellent impedance matching and attenuation constant values of the PPM/paraffin composites reveal their excellent potential to act as microwave absorbers. Therefore, PPM60/paraffin specimen is equipped with the optimal RL value of −57.57 dB and PPM80/paraffin sample is provided with the maximum *f*_E_ of 10.52 GHz. Based on these results, each of these composites exhibits great potential for high‐performance radar stealth applications, with their absorption properties tunable for various applications depending on the dip coating time

**Figure 5:**
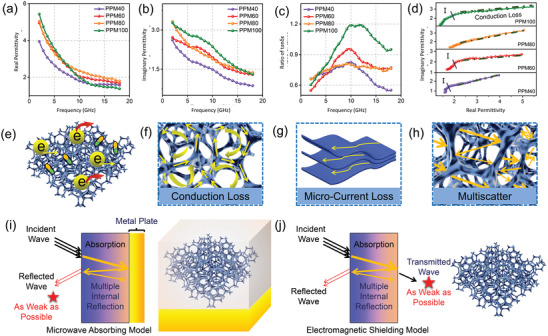
Frequency‐dependent electromagnetic parameters of PPM40, PPM60, PPM80, and PPM100/paraffin composites: a) real part of permittivity *ε′*, b) imaginary part of permittivity *ε″*, and c) dielectric loss tangent tan *δ*_*ε*_. d) Cole–Cole plots of PPM40/60/80/100 samples. e–h) Schematic illustration of probable microwave absorption mechanisms for the as‐prepared PPM foams. Schematic illustration of i) microwave absorbing model and j) electromagnetic shielding model of the 3D porous foams.

With the understanding that the samples prepared are composed of non‐magnetic components, measuring EM parameters should be sufficient to explain the relationship between dielectric loss and microwave absorption performance of these composites. As shown in **Figure** **5**a,b, both real permittivity (*ε*′) and imaginary permittivity (*ε*″) exhibit descending trends with increasing frequency, which can be explained by the frequency dispersion.^[^ ^57^ ^]^ Compared with other samples, PPM100/paraffin possesses the highest proportion of PEDOT:PSS. According to percolation theory, if the concentration of conductive filler is too high, the dielectric constant will decrease rapidly with increases in frequency.^[^ ^58^ ^]^ Therefore, the real part of the dielectric constant of PPM100/paraffin may decrease very quickly at high frequency. Looking at the imaginary part of the dielectric constant, PPM40/60/80/paraffin samples show clear evidence of dielectric relaxation behaviors (broad raised peak). This imaginary permittivity anomaly is predicted by the Debye relaxation theory as expressed in the following Equations:^[^ ^59^ ^]^

(6) $$\varepsilon \prime =\varepsilon_{\infty }+\left(\varepsilon_{s}-\varepsilon_{\infty }\right)/\left(1+\omega^{2}\tau^{2}\right)$$

(7) $$\varepsilon^{\prime \prime }=\left(\varepsilon_{s}-\varepsilon_{\infty }\right)\omega \tau /\left(1+\omega^{2}\tau^{2}\right)+\sigma /\omega \varepsilon_{0}$$

where *ω*, *τ*, *ε*_s_, *ε_∞_*, *σ*, and *ε*_0_ represent the angular frequency, the polarization relaxation time, the static permittivity, the relative dielectric permittivity at the high frequency limit, the alternative conductivity, and the dielectric constant in vacuum, respectively. From these equations, *ε*″ is regarded as the joint contributions from polarization relaxation and electrical conductivity. The observed dielectric relaxation behaviour in PPM40/60/100/paraffin composites probably originates from the relative inhomogeneity or aggregation of conductive components, resulting in inhomogeneous space charge distribution and forming interfacial polarization between PEDOT:PSS and melamine foam. Besides that, the dielectric loss tangent tan *δ_*ε*_* = *ε*″/*ε*′ was also calculated and plotted against frequency in Figure 5c.^[^ ^60^ ^]^ The resulting tan *δ_*ε*_* curves of the PPM40/60/100/paraffin composites each show a wide peak, which is consistent with the above phenomenon. Interestingly, the PPM80/paraffin sample peak is particularly gentle, with tan *δ_*ε*_* remaining within a comparatively small range. This may be ascribed to the fact that there is an optimum loading amount and distribution of PEDOT:PSS for electromagnetic wave transmission to occur. If the loading mass of PEDOT:PSS is too high, aggregation will result, while too low a loading mass will lead to poor conductivity, both of which will suppress electromagnetic wave transmission and attenuation. In order to further investigate the interplay between polarization loss and conduction loss, Cole–Cole plots (*ε*″ versus *ε*′ plots) have been provided in Figure 5d. The relationship between the dissipation (*ε*″) and storage (*ε*′) capacity can be established with the help of the static permittivity, *ε*_s_, and permittivity at infinite high frequency, *ε_∞_*:^[^ ^61^ ^]^

(8) $${\left(\varepsilon^{\prime }-\left(\varepsilon_{s}+\varepsilon_{\infty }\right)/2\right)}^{2}+{\left(\varepsilon^{\prime \prime }\right)}^{2}={\left(\left(\varepsilon_{s}-\varepsilon_{\infty }\right)/2\right)}^{2}$$

Thus, the Debye semicircles in Cole–Cole curves signify the relaxation process. For ease of observation, the amplified Cole–Cole plots are provided in Figure S15, Supporting Information. In the case of PPM40/60/100 paraffin samples, both polarization relaxation and conduction loss contribute to dielectric loss. The largely linear Cole–Cole curve of the PPM80/paraffin sample suggests that it possesses an appropriate mix of electrically conductive components which allows for efficient conduction loss and a uniform distribution of said components that bestows the material with a negligible level of relaxation loss (Figure 5d). Based on the above results, schematic illustrations of the probable EM attenuation mechanisms in play within the PPM/paraffin composites are shown in Figure 5e–h. Apart from the mentioned interfacial polarization, the conductive network in the composite may allow the presence of active hopping electrons and migrating electrons, contributing to conduction loss. Micro‐current loss results from the highly conductive PEDOT:PSS content. Another attenuation mechanism is multi‐scattering behavior, which comes about due to the 3D porous structure of the composite. To further understand the relevant mechanisms, electric field vector distribution on the upper surface of the 3D network structure and electric field distribution at 10 GHz were simulated using CST Studio Suite 2019 (PPM80 as an example). Figure S16a, Supporting Information, shows that surface current (little blue arrows) is concentrated on the wall of the 3D PPM80 porous network and is distributed along the direction of propagation (*y* direction) under TE (transverse electric wave) mode. From this, the overall physical length of the network directly affects the effective length of power transmission lines. Compared to a flat plate, the physical length of the network can be extended from a short straight route (the brown arrow) to a long zigzagging path (the red arrow). The effective electric length may be further increased by the abundant cross‐links in the 3D network structure, which reduces the effective wavelength along the Y transmission line, enhancing microwave absorption.^[^ ^62^ ^]^ Figure S16b, Supporting Information, shows the electric field distribution of the PPM80 sample at 10 GHz. The cavity inside the porous foam structure allows microwaves to travel into the material, effectively reducing reflection. It is quite clear that the electric field of the foam structure tends to decrease along the positive and negative *Z* directions, indicating that microwaves can be effectively attenuated by the material during their multi‐scattering propagation. Based on the metal back plane model (Figure 5i), improving input impedance matching at the air–material interface and the EM wave dissipation property of the component materials is necessary to develop better microwave absorbers.^[^ ^63^ ^]^ With all these mechanisms combined, the synthesized composites are undoubtedly excellent microwave absorbers that are highly suited for alleviating microwave pollution. When considering electromagnetic absorption, it is also advisable to consider the electromagnetic shielding capabilities of the composite (see in Figure 5j). This analysis will be carried out in a later part of this manuscript.

**Figure 6:**
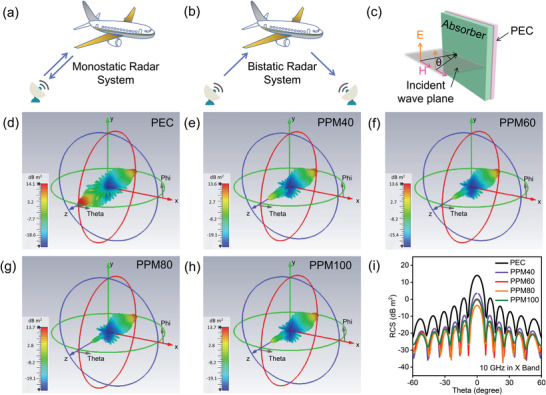
Schematic description of a) monostatic and b) bistatic radar systems. CST simulation results of c) PEC and d–h) PEC back plate covered with PPM40, PPM60, PPM80, and PPM100/paraffin composites, respectively. i) RCS simulated values of PEC and a sequence of PPM/paraffin/PEC composites within the scanning angle range from −60^o^ to 60^o^.

When examining materials for stealth applications, it has become clear that practical aspects such as radar target profiling and environmental conditions must be considered. Fortunately, computer simulation technology has shown great potential to allow research scholars to examine such aspects.^[^ ^64^ ^]^ In this work, the RCS of the as‐prepared conductive foam prototype/paraffin matrix composites in the far‐field response range have been simulated using CST software, providing information on the performance of absorbing materials under more realistic conditions. In this case, a model with size 20 × 20 × 5 cm^3^ was established using CST Studio Suite 2019 and the test frequency was set to 10 GHz. According to Equation (S16), Supporting Information, the *kα* value is larger than 10; thus, the target observed by radar system is in the optical region. It is desirable to minimize the RCS of objects to reduce the possibility of detection. Key equations describing radar and the calculation of RCS are provided in the Equations (S13)–(S16), Supporting Information. These equations are applicable for both monostatic and bistatic radar systems (**Figure** **6**a,b), which cover most available radar systems. Radar cross section (RCS, *σ*) can therefore be described using the equation below:

(9) $$\begin{array}{c} \sigma \left(m^{2}\right)=\lim_{R\to \infty }4\pi R^{2}{\left(\left|\frac{E_{s}}{E_{i}}\right|\right)}^{2}\underset{R\to \infty }{=\lim}4\pi R^{2}{\left(\left|\frac{H_{s}}{H_{i}}\right|\right)}^{2}=\lim_{R\to \infty }4\pi R^{2}\frac{S_{s}}{S_{i}} \end{array}$$

Here, *E*_s_ and *E*_i_ are the intensities of the scattered electric field and incident electric field, respectively. *H*_s_ and *H*_i_ stand for the intensities of the scattered magnetic field and incident magnetic field, and *S*_s_ and *S*_i_ represent the power density of the scattered field and incident field. In addition, as explained in the Supporting Information, the decibel square meter unit (dB m^2^) is typically used to measure radar cross section, where *σ* (dB m^2^) = 10 log *σ*(m^2^). The RCS values of the composites were therefore simulated, and the relevant 3D and 2D RCS simulation results are illustrated in Figure 6c–h. As can be observed from the 3D RCS simulation results in Figure 6c, a single PEC plate presents the largest RCS values out of those tested, which is clearly detrimental for stealth applications. Among the PPM/paraffin composites (Figure 6d–g), PPMP80/paraffin composites exhibit the lowest RCS values, showing the best stealth performance out of the group. To examine their performance further, the simulated RCS values within a scanning angle, *θ* range of −60° to 60° at 10 GHz (X band) of PEC and PPM/paraffincomposite‐covered PEC are shown in Figure 6h. Obviously, the RCS peak values decrease when *θ* is changed from 0° to ± 60°, proving scanning angles affect the displayed RCS. In the case of all samples, the obtained RCS value is highest at a scanning angle of 0^o^. The maximum RCS values for the PEC model and PPM40/60/80/100/paraffin‐covered PEC composites are 14.08, 3.64, −0.24, −3.6, and 0.15 dB m^2^, respectively. These results are consistent with the microwave absorption performance of each material illustrated in Figure 4. Based on these results, it can be seen that PPM80/paraffin/PEC reduces the RCS by 17.68 dB m^2^ compared to plain PEC, further demonstrating the excellent microwave attenuation ability of the PPM foam.^[^ ^65^ ^]^ As a rule, the factors influencing RCS values are: 1) structural and intrinsic properties of the target object, 2) frequency, 3) polarization of incident field and receiving antenna, and 4) the scanning angle.^[^ ^66^ ^]^ Given that factors apart from (1) are held constant (2,3) or controlled experimentally (4), it is quite clear that the highly porous and conductive structure of the PPM foam composites is responsible for the excellent stealth performance of the material. These lightweight functional materials demonstrate an excellent strategy to effectively modulate radar cross section in complex environments.

**Figure 7:**
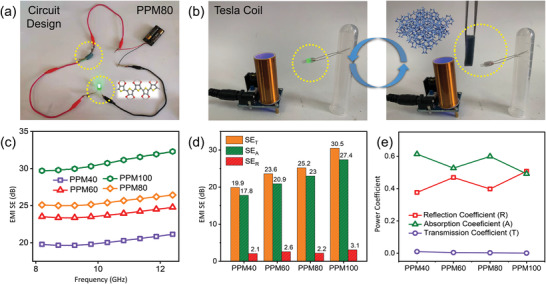
a) Circuit designation of an LED connected with the conductive PPM hybrid foam without conductive copper tape. b) Digital images of Tesla wireless transmission experiments for the 3D conductive PPM network. c) EMI shielding performances of a series of bulk PPM foams with the same thickness. d) Total shielding effectiveness (SE_T_) and shielding effectiveness by reflection (SE_R_) and by absorption (SE_A_) and e) power coefficients of the as‐prepared 3D PPM hybrid foams at the frequency of 10 GHz.

As discussed in previous sections, PPM foams possess outstanding electrical conductivity. In order to demonstrate this conductivity, a circuit containing an LED and two batteries connected with PPM80 hybrid foam without conductive copper tape has been designed. Due to its extremely high conductivity caused by its PEDOT content, there is no doubt that the PPM80 has low resistance (seen in Table S4, Supporting Information). As shown in **Figure** **7**a, the LED light in the circuit is brightly illuminated. Compared with PPM80 foam, the PPM40 sample possesses higher resistance; thus, a similar circuit containing a green light‐emitting diode and a power supply connected with the PPM40 specimen with conductive copper tape adhered on both ends was assembled. The brightness of the LED was found to increase when the PPM40 was subjected to the press of a finger (Figure S17, Supporting Information), which indicates that PPM40 has improved electrical conductivity under compression. With their excellent flexibility and electrical conductivity, PPM foams can be potentially utilized in piezoresistive sensors. As a proof‐of‐concept, a piece of PPM80 with conductive copper tape on both ends was connected to an electrochemical workstation to record its current response under varying compressive pressures. The real‐time current signals resulting from pressing the foam are shown in Figure S18, Supporting Information. When plotting *ΔI*/*I*_0_ against compressive stress on the PPM sponge, a linear relationship with a R^2^ value of 0.99332 is evident. As a result, the sensitivity (*S*) of this piezoresistive sensor can be defined as: *S* = (*ΔI*/*I*_0_)/*ΔP*.^[^ ^67^ ^]^ Here, *ΔI*/*I*_0_ = (*I_p_*−*I*_0_)/*I*_0_, where *ΔI* represents the difference between the real‐time current at a given strain (*I*_p_) and the initial current (*I*_0_), and *ΔP* stands for the change in applied pressure. Using this formula, one can find that the PPM sample has a sensitivity of 0.11 kPa^−1^. In addition to acting as EM absorbers, the PPM foams may also act as EMI shielding materials. Based on scattering parameters (S parameters, where S11 signifies the input reflection coefficient and S12 is the reverse transmission coefficient) tested using the waveguide method, the EMI shielding performance of the integral PPM foam prototypes with the dimension of 22.86 × 10.16 × 5 mm^3^ in Figure 7c may be calculated using the following equations:^[^ ^68^ ^]^

(10) $${\mathrm{SE}}_{R}=10\log\left(1/\left(1-{\left[S11\right]}^{2}\right)\right)$$

(11) $${\mathrm{SE}}_{A}=10\log\left(\left(1-{\left[S11\right]}^{2}\right)/{\left[S12\right]}^{2}\right)$$

(12) $${\mathrm{SE}}_{T}=10\log\left(1/{\left[S12\right]}^{2}\right)$$

Herein, SE_T_, SE_R_, and SE_A_ represent total shielding effectiveness and shielding effectiveness by reflection and by absorption, respectively. As expected, the EMI shielding effectiveness (SE) increases with increasing conductivity.^[^ ^69^ ^]^ The average EMI SE values in the X band of PPM foams with increasing dipping time are 20.16, 23.84, 25.51, and 30.80 dB for PPM40, 60, 80, and 100 respectively, showing excellent shielding properties that allow them to block 99–99.9% of incident microwaves.^[^ ^70^ ^]^ As all SE values are higher than 15 dB, the total EMI shielding effectiveness (SE_T_) may be approximated by the SE reflection (SE_R_) and SE absorption (SE_A_), ignoring the contribution from multiple reflections (SE_M_).^[^ ^71^ ^]^ The calculated values are shown in Figure 7d, which makes it clear that SE_A_ is higher than SE_R_ at 10 GHz. This suggests that the PPM foams shield against EM radiation mainly through absorption. In addition, the EMI shielding mechanisms at the frequency of 10 GHz may also be examined using the power coefficients of absorption (*A*), reflection (*R*), and transmission (*T*). As a rule, *A* + *R* + *T* = 1, where *T* = |S_21_|^2^ and *R* = |S_11_|^2^.^[^ ^72^ ^]^ The corresponding power coefficients vary with the dipping time of MF in the conductive PEDOT:PSS solution, which can be clearly seen in Figure 7e. For PPM40/60/80 specimens, *T* values tend to be 0 and *A* values are larger than *R* values, so absorption likely plays a dominant role in their EMI shielding mechanisms. In the case of PPM100, *T* value is 0 and there seems to be little difference between *A* and *R*, so it appears that absorption and reflection contribute roughly equally. The overall dominance of the absorption mechanism may be ascribed to the PPM foams’ extremely high electrical conductivity and 3D network with plentiful heterogeneous interfaces, that allow for conduction loss and multiple scattering. As the electromagnetic shielding model shows (Figure 5j), if there exhibits almost no transmitted electromagnetic waves, the conductive PEDOT:PSS@melamine composite can be utilized for efficient EMI shielding. To demonstrate the practical ability of the PPM foams to act as an electromagnetic shielding material, a Tesla coil was used to light a green bulb (Figure 7b) and blocked using a piece of PPM foam (Figure 7f). The relevant mechanism is illustrated in Figure S19, Supporting Information. It can be seen that the PPM foam is capable of blocking the wireless transmission of energy from the Tesla coil to the green LED, preventing it from lighting up. In addition, Videos S11 and S12, Supporting Information, present a similar experiment using two custom‐assembled Tesla coils and neon bulbs, in which the neon bulbs show increased brightness when the PPM foam is moved away. These demonstrations clearly show that the PPM foams are effective for electromagnetic shielding.

## 3 Conclusion 

In summary, lightweight (density ranges from 0.1220 to 0.1694 g cm^−3^), mechanically elastic (the maximum stress value of 34.5, 37.3, 57.7, and 82.0 kPa) and thermally‐insulating (low thermal conductivity from 0.053 to 0.025 W m^−1^·K^−1^) PPM hybrid foams for high‐performance infrared‐radar compatible stealth were synthesized via a facile pretreatment of MF and a subsequent impregnation process. With an increase in dipping time (from 40 to 100 min), electrical conductivity values increase from 0.58 to 2.16 S m^−1^, respectively. Accordingly, IR emissivity values decrease from 0.872 to 0.788 in the 3–5 µm band and from 0.994 to 0.757 in the 8–14 µm band, which may help inspire further progress in developing effective infrared stealth materials. Furthermore, a minimum RL value of −57.57 dB could be achieved by the PPM60/paraffin sample and a maximum effective bandwidth of 10.52 GHz could be reached by the PPM80/paraffin sample, which could be attributed to the sample's excellent microwave attenuation ability and appropriate impedance matching. In addition, the maximum RCS values for PPM80/paraffin covered PEC composites can be reduced from 14.08 to −3.6 dB m^2^ compared to PEC alone, indicating that PPM/paraffin composites in this work have great potential for protecting devices from detection. Besides the outstanding microwave absorbing performance, the hybrid foams are also excellent EMI shielding materials with average EMI SE values ranging up to 30.80 dB for the PPM100 foam. The PPM foams’ simple and efficient preparation method and highly versatile nature grants them great potential to satisfy applications in both infrared and microwave stealth applications.

## 4 Experimental Section

### Materials

Melamine foam (MF) and PEDOT:PSS (PH1000) were bought from Sichuan Chemical Co., Ltd and Heraeus Deutschland GmbH Co KG, separately. Sodium hydroxide (NaOH), dimethyl sulfoxide (DMSO), and dodecylbenzene sulfonic acid (DBSA) was purchased from Nanjing Chemical Reagent Co., Ltd.

### Preparation of PEDOT:PSS@Melamine Hybrid Foams

The pretreatment of melamine foams was divided into three steps. Step I: A series of melamine sponges were cut into cylindrical pieces or rectangle pieces with different dimensions for further characterization. Step II: The tailored melamine foams were cleaned by ultrasonic washing using excessive amount of deionized water and ethyl alcohol for 30 min each. Step III: In order to endow the surface of all the 3D porous samples with hydrophilicity, these specimens were totally immersed in sodium hydroxide (NaOH) solution with the concentration of 5 mol L^−1^, and the reaction was kept at 65 °C for half an hour. The hydroxyl‐terminated melamine foams were dried at 60 °C in a vacuum drying chamber for several hours, which were denoted as (pristine) MF. Subsequently, the impregnation process was carried out. First, a specific PEDOT:PSS solution was fabricated by dissolving 0.5 g DMSO and 0.1 g dodecylbenzene sulfonic acid (DBSA) in 9.5 g PH1000 PEDOT:PSS dispersion for 20 min. Afterward, MF specimens were completely dipped in the above solution for 40, 60, 80, and 100 min, respectively. After that, excessive liquid was squeezed out of these 3D hybrid foams. Finally, the corresponding conductive PEDOT:PSS@melamine composite was washed by deionized water and placed in a vacuum oven at 60 °C for 6 h, which can be named as PPM40/60/80/100 (PPM samples). The specific PEDOT:PSS solution had also been dried at 60 °C, which can be named as PP for thermal conductivity test.

### Morphology and Chemical Structure Characterization

The FE‐SEM images were taken on a Hitachi S4800 type microscope to observe microscopic morphology and structure of PEDOT:PSS, MF and PPM samples. To carry out the mercury intrusion method, a MicroActive AutoPore V 9600 2.03.00 device was adopted to obtain pore size and porosity information of lightweight MF specimen and PPM hybrid foams. Characteristic functional groups of the as‐obtained 3D PPM composites were recorded using a Nicolet 5700 FT‐IR (Fourier Transform Infrared) spectrometric analyzer. The difference in elemental composition between MF and PPM samples was analyzed using an X‐ray photoelectron spectrometer (XPS, PHI 5000 VersaProbe) with an Al K*α* X‐ray source at 150 W.

### Thermal Insulation and Infrared Stealth Test

Infrared thermal imaging photos with the information of different foams’ (length: 30 mm; width: 28 mm; thickness: 10 mm) surface temperature and infrared stealth property were taken by a TVS‐2000MK type infrared imaging device. For further investigating the heat insulation performance, thermal conductivity of all samples (2.5 × 2.0 × 1.1 cm^3^) was measured at 80 °C by a Hot Disk TPS2500S based on the ISO 22007‐2 standard. Specific infrared emissivity values in 3–5 µm and 8–14 µm wavelengths were also recorded (by an IR‐2 dual‐band emissivity measuring instrument) as additional evidence to verify the infrared stealth capability of the final PPM samples (diameter: 5 cm, thickness: 1 cm). According to GB/T2406.2‐2009/ISO:4589‐1/ISO:4589‐2/ASTM D2863 standards, the limiting oxygen index (LOI) of the sample (8 × 1 × 0.4 cm^3^) was tested in a FTT0077 oxygen index meter.

### Electrical Resistance and Sensing Performance Test

Electrical conductivity of the intact PPM hybrid foams was visually validated via design of electric circuit with a light‐emitting diode (using PPM40) and self‐assembly Tesla wireless transmission device with a neon bulb (using PPM80). Besides that, bulk resistance values test of PPM foams (2.5 × 1.0 × 1.0 cm^3^) were performed via a ZC‐90 resistance test apparatus. According to the relationship between electrical resistivity and conductivity, there shows the following calculation formulas *ρ* = *RS*/*L* and *σ* = *1*/*ρ*, where *ρ* represents resistivity (Ω·m), *R* signifies resistance values (Ω), *S* means cross section area (1.0 × 1.0 cm^2^) of the test sample, *L* stands for the length (0.025 m) of the foam, and *σ* is the electrical conductivity (S·m^−1^). With regard to the mechanical performance, a universal testing machine (CMT5105) was utilized to compare the compressive strength of the as‐prepared 3D PPM hybrid foams with the same dimension of 2.5 × 2.0 × 1.1 cm^3^. After the connection of the PPM foam sensor (2.5 × 1 × 1 cm^3^) to a CHI 660E type electrochemical workstation, its piezoresistive sensing properties under various compressive pressures were analyzed using real‐time current response.

### Electromagnetic Parameters Test

Electromagnetic parameters including scattering parameters (S11 and S21) and complex permeability and permittivity were measured using an advanced vector network analyzer (VNA, Agilent PNA N5244A) with internal setup of the formula calculation procedure. Based on the waveguide method, integral foam prototypes with the dimension of 22.86 × 10.16 × 5 mm^3^ were used to test S parameters for further calculating EMI shielding effectiveness (EMI SE) and transmission (*T*), reflection (*R*), and absorption (*A*) coefficients in X band. Due to excellent electrical conductivity of PPM, paraffin needed to be added to optimize impedance matching performance, so complex permeability and permittivity were tested using the PPM/paraffin composite form. Based on the coaxial‐line method, 80 wt% intact foam prototypes cut with the column of 7.00 × 3.04 × 2.00 mm^3^ (outer diameter × inner diameter × thickness) were mixed with 20 wt% paraffin to obtain typical toroidal rings.

### Simulation

Commercial software of CST Studio Suite 2019 was used. Electric field vector distribution on the upper surface of the 3D network structure and electric field distribution at 10 GHz were simulated using computer simulation technology (CST). Due to computational constraints, a simplified 3D porous foam model was established to reduce the computational burden. It is well‐known that CST can also be applied for simulating radar cross section (RCS) of the as‐prepared hybrid foams under actual far‐field response. In this work, CST was also accepted to establish 20 × 20 × 5 cm^3^ models and complete the RCS simulation research.

### Statistical Analysis

Sample surface temperature was determined by taking the average of five distinct points. Infrared emissivity value of each sample was the average of six tests. The limiting oxygen index value was confirmed by taking measurements on 15 pieces of each sample. Two different Tesla coils were assembled to shoot the wireless transmission video presentation. Electrical resistance was measured using three different pieces of each foam. Mechanical and sensing performance were tested three times, and one of the three similar sets of data was chosen as the final data to draw figures. Electromagnetic parameters were also tested using three different samples and one of the three similar sets of data was chosen.

## Conflict of Interest

The authors declare no conflict of interest.

## Supporting information

Supporting Information

Supplemental Video 1

Supplemental Video 2

Supplemental Video 3

Supplemental Video 4

Supplemental Video 5

Supplemental Video 6

Supplemental Video 7

Supplemental Video 8

Supplemental Video 9

Supplemental Video 10

Supplemental Video 11

Supplemental Video 12

## Data Availability

The data that support the findings of this study are available from the corresponding author upon reasonable request.
