# Supplementary material for: A Lightweight, Elastic, and Thermally Insulating Stealth Foam With High Infrared‐Radar Compatibility
Source: Adv Sci (Weinh). 2022 Oct 26;9(35):2204165. doi: 10.1002/advs.202204165 (PMC9762302; doi:10.1002/advs.202204165)
Supplement: Supplementary file 1 — Supporting Information [file ADVS-9-2204165-s007.pdf]

ADVANCED SCIENCE
Open Access

Supporting Information

for *Adv. Sci.*, DOI 10.1002/advs.202204165

A Lightweight, Elastic, and Thermally Insulating Stealth Foam With High Infrared-Radar Compatibility

Weihua Gu, Samuel Jun Hoong Ong, Yuhong Shen, Wenyi Guo, Yiting Fang, Guangbin Ji and Zhichuan J. Xu**

Copyright WILEY-VCH Verlag GmbH & Co. KGaA, 69469 Weinheim, Germany, 2018.

Supporting Information

A Lightweight, Elastic, and Thermally Insulating Stealth Foam with High Infrared-Radar Compatibility

Weihua Gu, Samuel Jun Hoong Ong, Yuhong Shen, Wenyi Guo, Yiting Fang, Guangbin Ji, Zhichuan J. Xu**

Contents

1. Density of a series of three-dimensional PPM foams after different dipping time and relevant gravimetric loading capacity. (Table S1)

2. Average pore size and porosity information of MF specimen and 3D PPM foams. (Table S2)

3. The limiting oxygen index (LOI) of MF specimen and 3D PPM foams. (Table S3)

4. Electrical conductivity of the pristine melamine foam and the as-prepared PPM foams. (Table S4)

5. The specific EM wave absorption performance (SMAP) of the as-prepared PPM/paraffin composites. (Table S5)

6. Comparison of PEDOT:PSS aerogels in recent literature and PEDOT:PSS@melamine composite. (Table S6)

7. SEM images of the pristine melamine foams before pretreatment and after pretreatment. (Figure S1)

8. Magnified SEM images of PPM40, PPM60, PPM80, and PPM100 foams. (Figure S2)

9. Mercury intrusion and extrusion curves of MF, PPM40, PPM60, PPM80, PPM100. (Figure S3)

10. Digital image of bearing capacity of PPM80 sample. (Figure S4)

11. Fatigue test of MF at 60% strain for 200 cycles. (Figure S5)

12. Humid aging test of MF and PPM40/60/80/100 under constant temperature (23 ^{o}C) and variable humidity. (Figure S6)

13. Thermal infrared images of pristine MF, PPM40, PPM60, and PPM100 sample captured from 0 to 10 min. (Figure S7-10)

14. Side view of thermal infrared images of PPM80 sample. (Figure S11)

15. Thermal conductivity of all samples and thermal transfer mechanism of the 3D porous network. (Figure S12)

16. The upward trend of electrical conductivity of the PPM samples with the increase of dipping time. (Figure S13)

17. Infrared emissivity plots of pristine MF and PPM40/60/80/100 hybrid foams in the infrared band of 3-5 μm and 8-14 μm. (Figure S14)

18. Derivation procedure of delta-function.

19. Cole-Cole plots of PPM40, PPM60, PPM80, and PPM100 samples. (Figure S15)

20. Simulation of electric field vector distribution and electric field distribution of the porous foam sample. (Figure S16)

21. Radar equation and radar cross section (RCS) simulation theroy.

22. Electric circuit experiment. (Figure S17)

23. Sensing performance. (Figure S18)

24. Tesla wireless transmission device and relevant mechanisms. (Figure S19)

Table S1. Density of a series of three-dimensional PPM foams after different dipping time and relevant gravimetric loading capacity.

Sample	M_0 (g)	M_S (g)	M_L (g)	Volume (cm^3)	Density after dipping (g/cm^3)	Q (g/g)
PPM40	0.0569	0.6712	0.6143	5.5	0.1220	10.80
PPM60	0.0568	0.8145	0.7577	5.5	0.1377	13.34
PPM80	0.0586	0.8679	0.8093	5.5	0.1471	13.81
PPM100	0.0578	0.9893	0.9315	5.5	0.1694	16.12

Herein, M_0 represents mass of PPM samples before dipping, M_S means mass of PPM samples after dipping, M_L signifies loading mass of PEDOT:PSS, and Q is the gravimetric loading capacity of PEDOT:PSS. All specimens were cut as 2.5 × 2.0 × 1.1 cm^3 and then weighed by an analytical electronic balance. As is known to all, density is obtained via dividing mass by volume. In addition, the gravimetric loading capacity (Q, g/g) of the PPM foams was calculated as: [1]

$$Q = \frac{M_S - M_0}{M_0} \qquad \text{(S1)}$$

Table S2. Average pore size and porosity information of MF specimen and 3D PPM foams.

Sample	Average pore size (μm)	Porosity (%)
MF	198.003	98.89
PPM40	137.447	92.67
PPM60	130.127	95.84
PPM80	129.840	95.67
PPM100	124.443	92.67

Table S3. The limiting oxygen index (LOI) of MF specimen and 3D PPM foams.

Sample	LOI (%)
MF	33.59
PPM40	31.25
PPM60	30.90
PPM80	26.30
PPM100	25.19

Table S4. Electrical conductivity of the pristine melamine foam and the as-prepared PPM foams.

Sample	R (Ω)	S (m^2)	L (m)	ρ (Ω·cm)	σ ($S\cdot m^{-1}$)
MF	∞	0.01^2	0.025		
PPM40	428	0.01^2	0.025	1.71	0.58
PPM60	233	0.01^2	0.025	0.932	1.07
PPM80	189	0.01^2	0.025	0.756	1.32
PPM100	116	0.01^2	0.025	0.464	2.16

Table S5. The specific EM wave absorption performance (SMAP) of the as-prepared PPM/paraffin composites.

Sample	Density of the foam/paraffin composites (g/cm^3)	SMAP (dB·cm^3/g) at 3.9 mm	SMAP (dB·cm^3/g) at 4.25 mm	SMAP (dB·cm^3/g) at 5 mm
PPM40/paraffin	0.1220	2000.44	2264.93	2556.81
PPM60/paraffin	0.1377	2891.34	2983.33	2854.12
PPM80/paraffin	0.1471	3262.02	3259.42	2654.74
PPM100/paraffin	0.1694	2709.35	2643.52	2196.38

The specific EM wave absorption performance SMAP: the SMAP value is defined as the ratio of the average RL value to the product of density and thickness in the measured frequency range. The SMAP value is calculated by the following equation: [2]

$$\text{SMAP}\ (\text{dB}\cdot\text{cm}^3/\text{g}) = \frac{\left|\int_{f_a}^{f_b}(\text{RL})df\right|}{(f_b - f_a)\times d\times \rho_{bulk}} \qquad \text{(S2)}$$

Where *fa* and *fb* represent the start frequency (2 GHz) and stop frequency (18 GHz), RL is the reflection loss values of the samples in the measured frequency range, *d* stands for the thickness of the foam/paraffin composite, and ρ_{bulk} is the bulk density (g/cm^3) of the test sample. In this case, the test sample is composed of 80 wt % PPM foam and 20 wt % paraffin, and the volume is fixed. Thus, ρ_{bulk} can be obtained via the density after dipping in Table S1 dividing 0.8, and the calculated results are given in Table S5.

Table S6. Comparison of PEDOT:PSS aerogels in recent literature and PEDOT:PSS@melamine composite.

Materials	Preparation method	Key point of the research	Reference
MXene/PEDOT:PSS aerogel	Electrogelation	The aerogel can be assembled into pressure sensors for wearable physical monitoring and high-resolution sensor microarrays for robotic tactile sensing.	[3]
MXene/PEDOT:PSS aerogel	Freeze-drying	Providing a practical approach for designing and fabricating lightweight absorption-dominated electromagnetic interference shielding materials.	[4]
Ag NW/PEDOT:PSS/PI aerogel	Freeze-drying	Finding a balance between stability, sensitivity, and sensing range, especially under the low-pressure range in piezoresistive sensing field.	[5]
PEDOT:PSS/PBP aerogel	Freeze-drying	Paving the way for improving the flexibility of a well-formed aerogel with cellular structures.	[6]
Cellulose nanocrystal-PEDOT:PSS aerogel	3D freeze printing	Using a novel additive manufacturing method to fabricate aerogel.	[7]
PEDOT:PSS aerogel	Freeze-drying; dipping method; supercritical CO_2 treatment	The structure and thermoelectrical performance of PEDOT:PSS aerogels treated with secondary doping by the dipping and supercritical CO_2 methods.	[8]
rGO/PEDOT:PSS aerogel	Hydrothermal reaction followed by acid treatment	High performance for supercapacitor electrodes.	[9]
PEDOT:PSS/polyimide aerogel	Freeze-drying	Solving the trade-off between robust mechanical properties and elastic-responsive conductivity.	[10]
PDMS-infiltrated graphene-PEDOT:PSS aerogel	Freeze-drying	Combining novel impedance method with the as-prepared sensor to obtain the simultaneous determination of multiple stimuli.	[11]
Bacterial cellulose nanofibril-PEDOT/PSS aerogel	Freeze-drying	Enhancing rheological properties and conductivity of bacterial cellulose hydrogels and aerogels through complexation with metal ions and PEDOT/PSS.	[12]

PEDOT:PSS/GO aerogel	Freeze-drying	Utilizing thermal evaporation for electrical energy harvesting.	[13]
PEDOT:PSS@melamine foam	Facile dipping method	Providing significant guidance for the design and fabrication high performance infrared-radar compatible stealth composites and rationally simulating the actual radar cross section and electric field distribution via smart CST technology.	This work

To highlight the merits of our work, a comparison of preparation method and key point of this work and works reporting similar PEDOT:PSS aerogels is shown in Table S6. It can be seen that most previous works use the freeze-drying method to synthesize their aerogels. As in the name, freeze-drying requires the precursor solution to freeze and then dry. The fact that drying is necessary implies that the aerogel/foam prepared in this manner would undergo rapid deterioration in real-world conditions involving high humidity. However, the PPM foam in this work shows good stability in humid environments. The results of a humid aging test can be seen in Figure S6 below. Among the previous works, there are also some novel preparation methods, such as 3D freeze printing. However, these methods are fairly complicated, and the simple dipping method in our work is shown to be sufficient to prepare samples with good performance. The simplicity of our method also potentially allows reduced energy usage. In addition, most of the previous works focus on sensing performance, tuning conductivity, and electromagnetic interference shielding. While these applications are highly necessary and demonstrate the effectiveness of PEDOT:PSS, there has been little study of infrared radar compatibility with foams/aerogels composed of PEDOT:PSS. In this manner, our work helps to fill this gap and provide significant guidance for the design and fabrication of multi-spectral compatible stealth materials and devices. It is also worth noting that our work demonstrates the simulation of radar cross section (RCS) under far-field environment, the electric field vector distribution on the upper surface of the 3D PPM foam, and electric field distribution at 10 GHz using smart CST technology, which is rare in the available literature. This work may give inspiration and set a paradigm for deeper explanations of electromagnetic attenuation mechanisms using CST simulations.

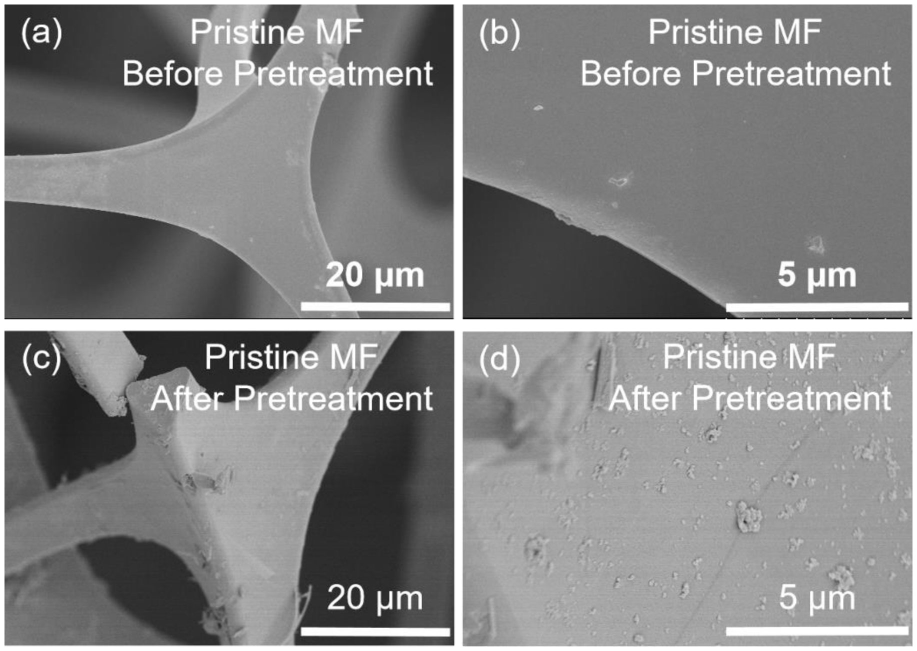

Figure S1. SEM images of the pristine melamine foams a, b) before pretreatment and c, d) after pretreatment.

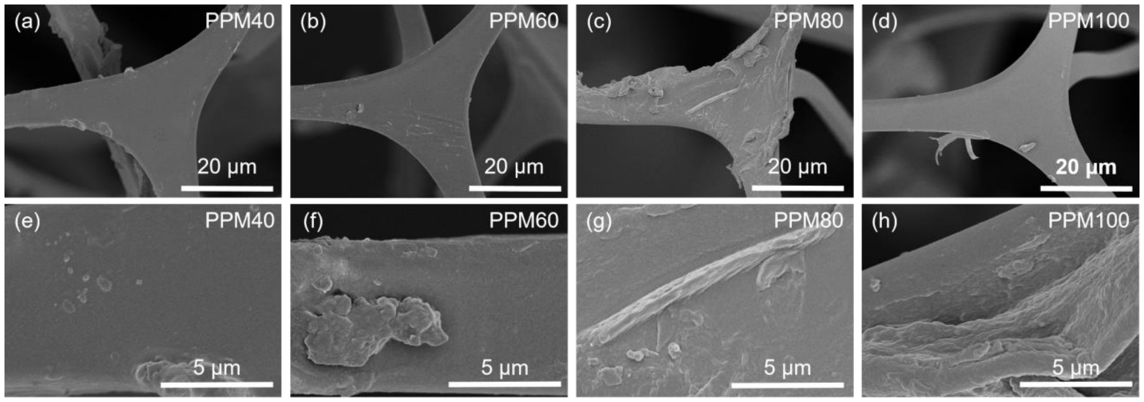

Figure S2. Magnified SEM images of a, e) PPM40, b, f) PPM60, c, g) PPM80, and d, h) PPM100 foams, respectively.

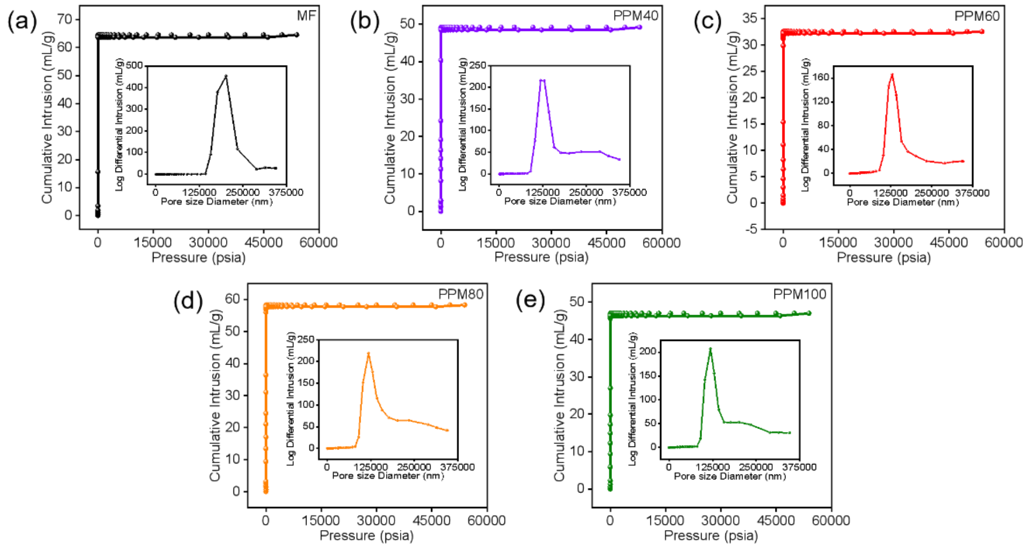

Figure S3. Mercury intrusion and extrusion curves of a) MF, b) PPM40, c) PPM60, d) PPM80, e) PPM100, respectively. The insets present the relevant pore size distributions.

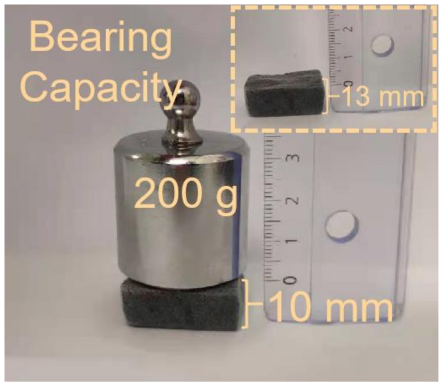

Figure S4. Digital image of bearing capacity of PPM80 sample.

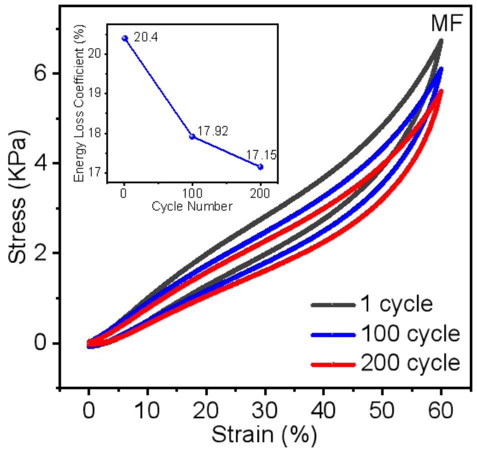

Figure S5. Fatigue test of MF at 60% strain for 200 cycles. Inset: energy loss coefficients over 200 cycles.

Humid aging test:

Humid aging performance of a series of PPM foams was carried out using a SETH-Z-032L type high-and-low temperature humid heat test chamber. The experiment was carried out with a constant temperature of 23 ^{o}C, and humidity settings in the following manner: (1) 25%RH (relative humidity) for 5 min; (2) 25%RH for 5 min, then 60%RH humidity for 5 min; (3) 25%RH for 5 min then 0%RH for 5 min, and finally 95%RH for 5 min. The samples were monitored for changes in appearance such as discoloration, spotting, dissolution and shedding using digital photos.

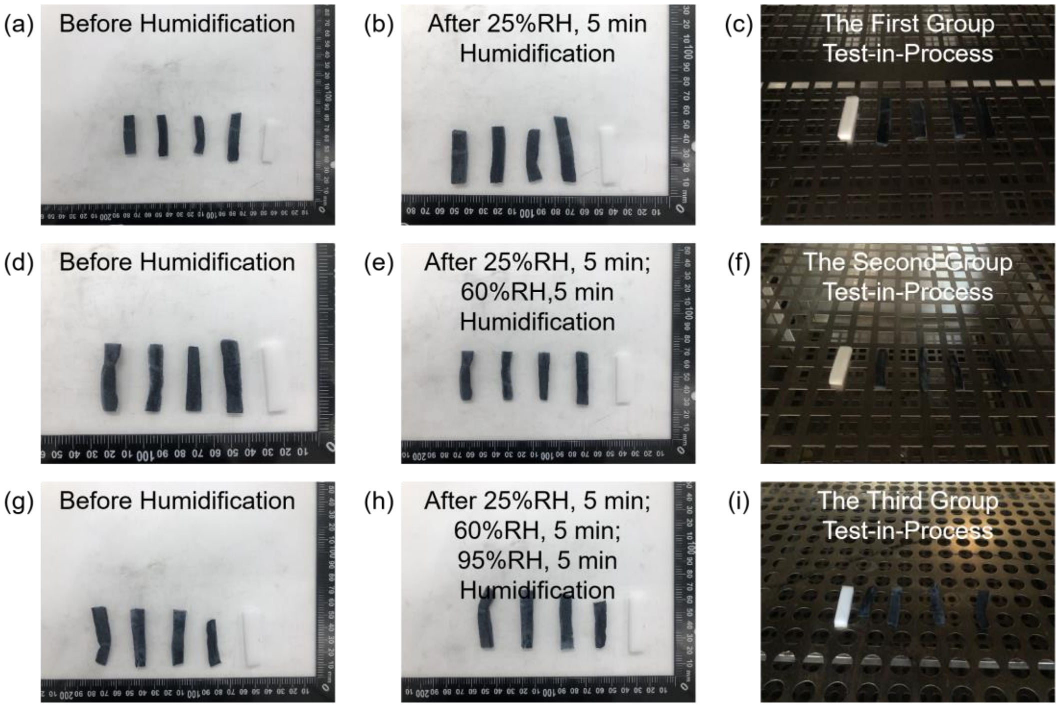

Figure S6. Humid aging test of MF and PPM40/60/80/100 under constant temperature of 23 ^{o}C and (a-c) 25%RH for 5 min; (d-f) 25%RH for 5 min and then 60%RH for 5 min; (g-i) 25%RH for 5 min, then 60%RH for 5 min, and finally 95%RH for 5 min.

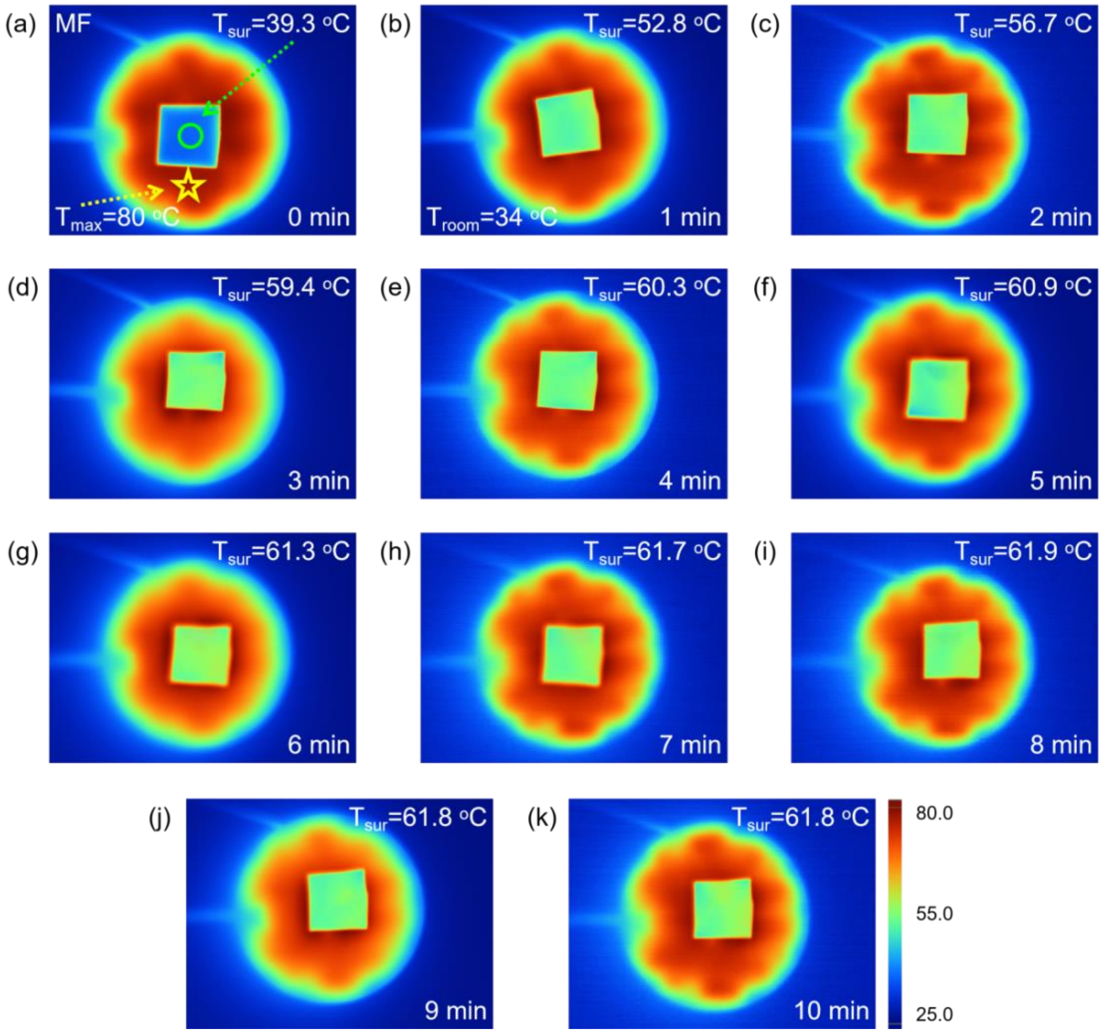

Figure S7. Thermal infrared images of pristine MF sample captured from 0 to 10 min.

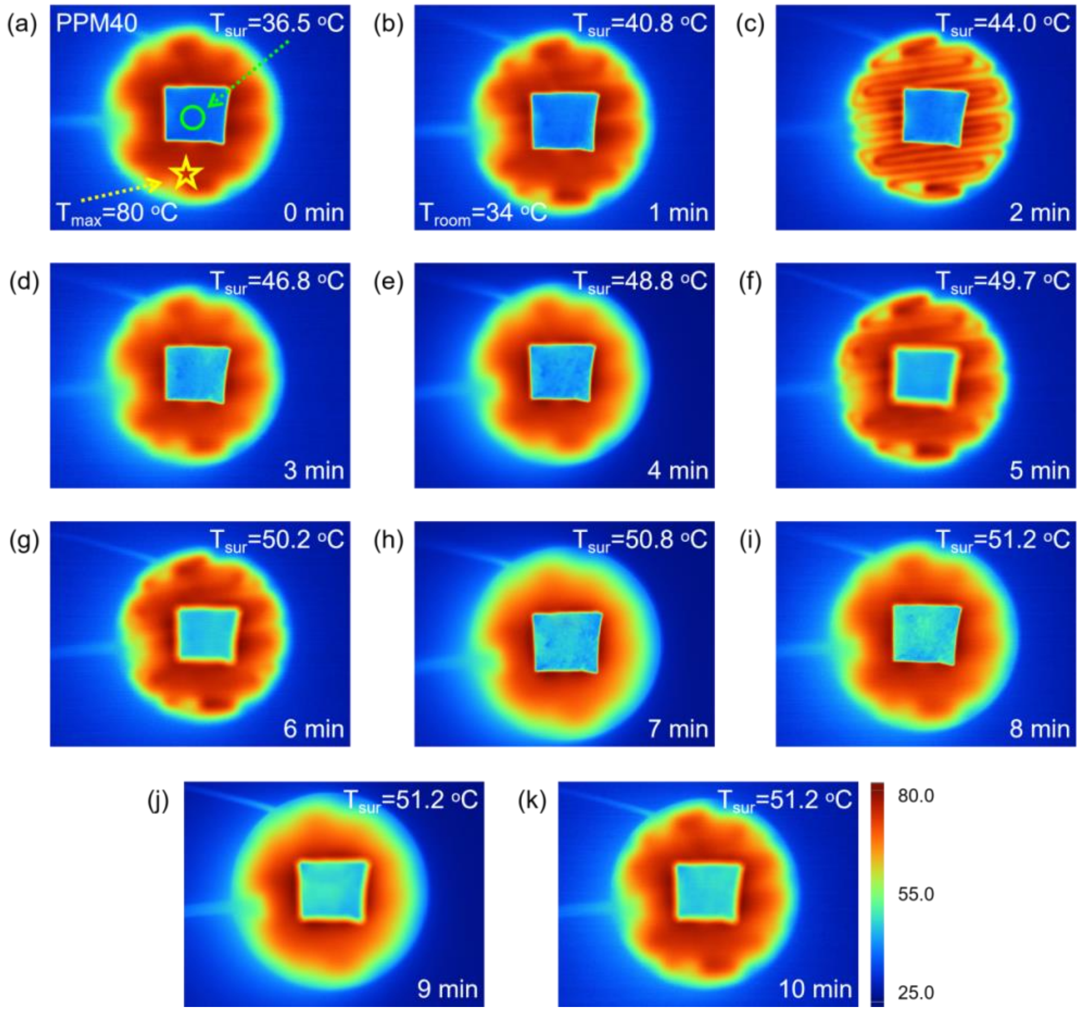

Figure S8. Thermal infrared images of PPM40 sample captured from 0 to 10 min.

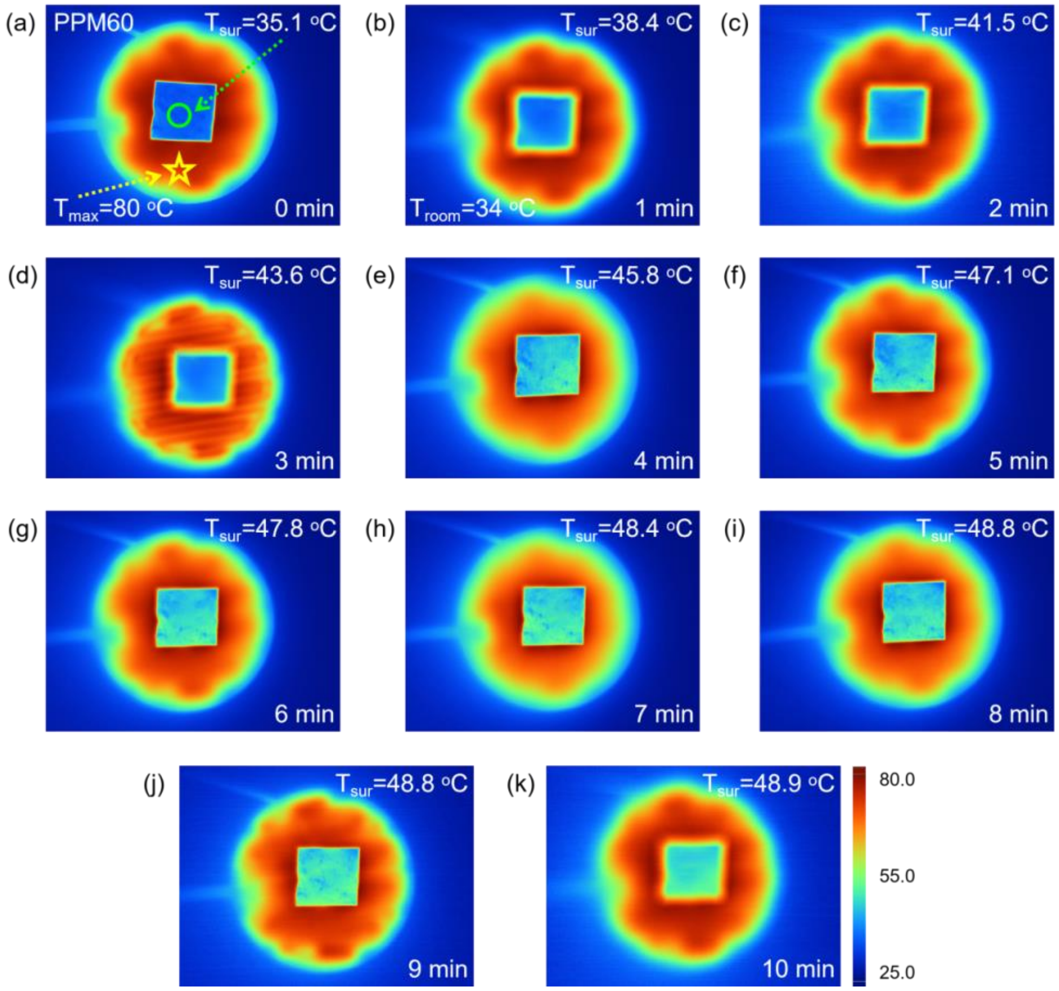

Figure S9. Thermal infrared images of PPM60 sample captured from 0 to 10 min.

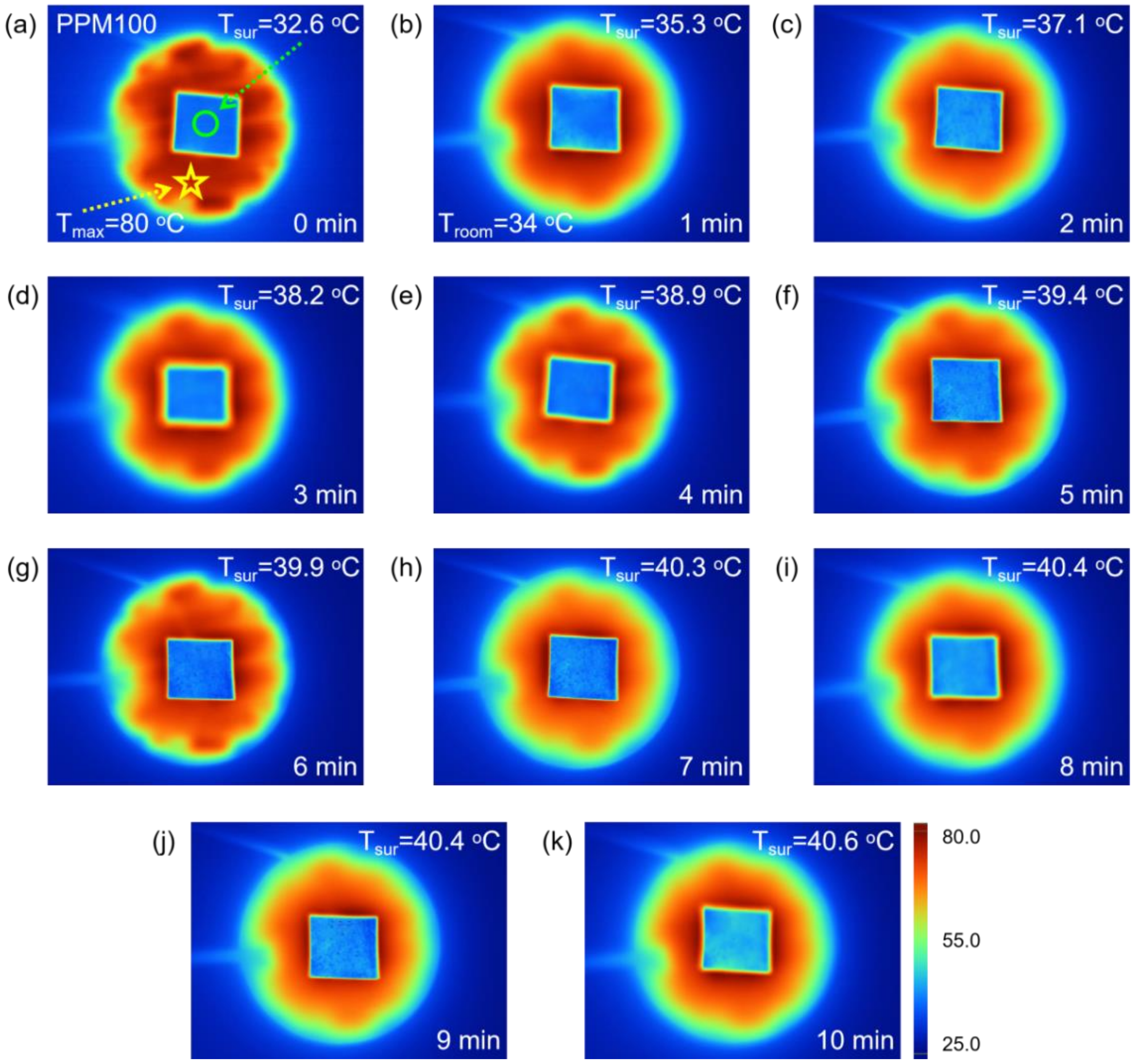

Figure S10. Thermal infrared images of PPM100 sample captured from 0 to 10 min.

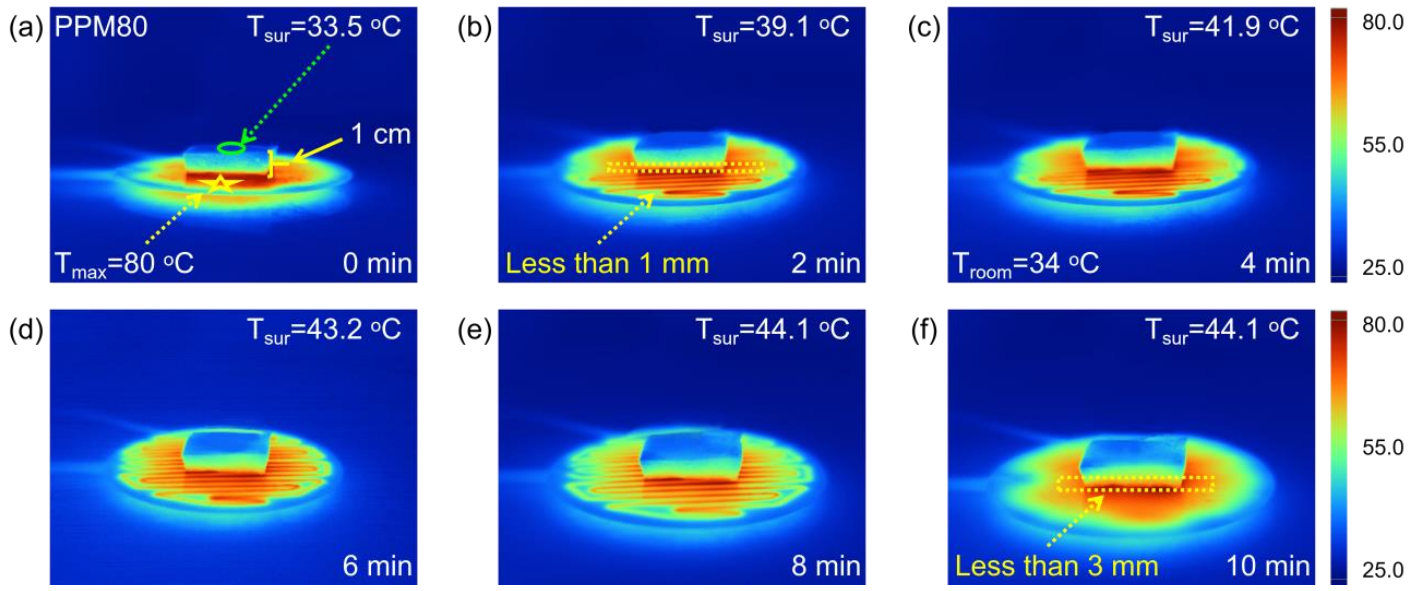

Figure S11. Side view of thermal infrared images of PPM80 sample.

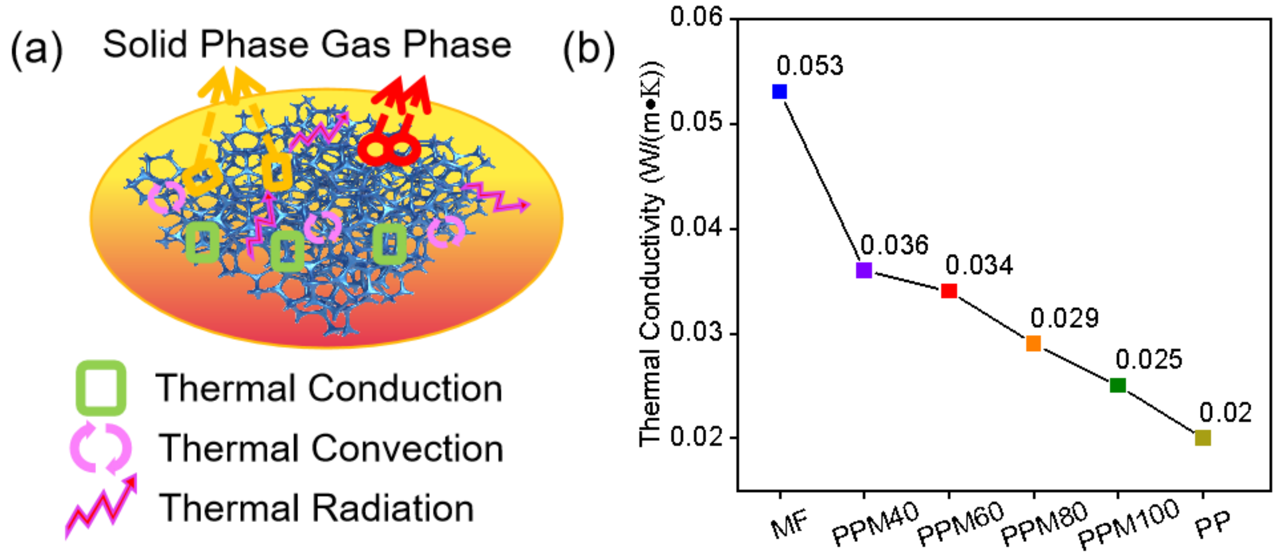

Figure S12. (a) Schematic illustration of the thermal transfer mechanism for the 3D porous network. (b) Thermal conductivity of MF, PPM40/60/80/100 and PP at the test temperature of 80 °C.

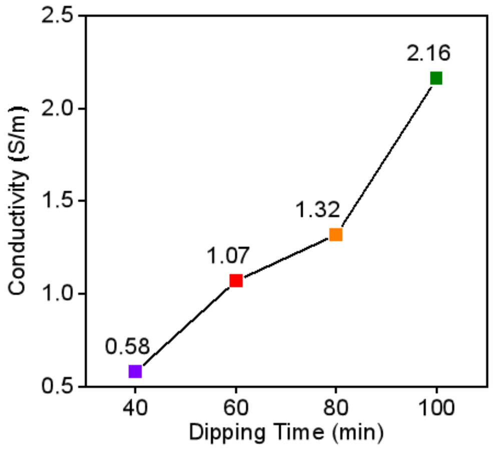

Figure S13. The upward trend of electrical conductivity of the PPM samples with the increase of dipping time.

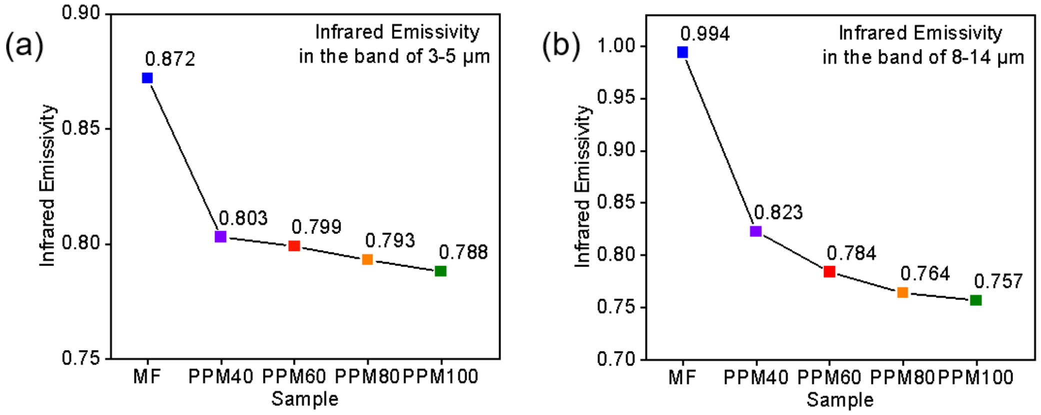

Figure S14. Infrared emissivity plots of pristine MF and PPM40/60/80/100 hybrid foams in the infrared band of (a) 3-5 μm and (b) 8-14 μm.

Derivation procedure of delta-function:

According to the well-known formulas about the input impedance Z_{in} at the air-material interface and reflection loss values of the microwave absorbers: [14,15]

$$Z_{in} = Z_M/Z_0 = (\mu_r/\varepsilon_r)^{1/2}\tanh[j(2\pi fd/c)(\mu_r\varepsilon_r)^{1/2}] \tag{S3}$$

$$RL = 20log|(Z_{in}-1)/(Z_{in}+1)| \tag{S4}$$

Where symbols are detailed explained in the manuscript, so there is no need to repeat them again. When Z_{in} approaches 1, RL values will reach -∞, namely, there exhibit almost no reflection. Thus, formula (2) can be transformed into the following equation:

$$1/\tanh[j(2\pi fd/c)(\mu_r\varepsilon_r)^{1/2}] = (\mu_r/\varepsilon_r)^{1/2} \tag{S5}$$

Both the left and the right sides of the equation possess frequency-dependent complex permeability μ_r and permittivity ε_r, thus, the above equation is a complex function. In order to acquire a real function, the real and imaginary parts of the relative permeability and permittivity should be substituted into the above formula. On the basis of trigonometric function and complex equation theory, equations can be derived as the follows: [16]

$$\cos\theta = \frac{\mu'\cos\delta_e - \varepsilon'\cos\delta_m}{\mu'\cos\delta_e + \varepsilon'\cos\delta_m} \times \cosh\left(\frac{4\pi fd\sqrt{\mu'\varepsilon'}\sin[(\delta_e+\delta_m)/2]}{c\sqrt{\cos\delta_e\cos\delta_m}}\right) \tag{S6}$$

$$\sin\theta = \tan\frac{\delta_e+\delta_m}{2}\sinh\left(\frac{4\pi fd\sqrt{\mu'\varepsilon'}\sin[(\delta_e+\delta_m)/2]}{c\sqrt{\cos\delta_e\cos\delta_m}}\right) \tag{S7}$$

Herein, θ, δe, and δm can be expressed as:

$$\theta = \frac{4\pi fd}{c}\sqrt{\mu'\varepsilon'}\frac{\cos(\delta_e/2+\delta_m/2)}{\sqrt{\cos\delta_e\cos\delta_m}} \tag{S8}$$

$$\delta_e = \arctan\frac{\varepsilon''}{\varepsilon'} \tag{S9}$$

$$\delta_m = \arctan\frac{\mu''}{\mu'} \tag{S10}$$

For simplification, we can define K and M as the follows:

$$K = \frac{4\pi}{c}\sqrt{\mu'\varepsilon'}\frac{\sin[(\delta_e+\delta_m)/2]}{\cos\delta_e\cos\delta_m} \tag{S11}$$

$$M = \frac{4\mu'\cos\delta_e\varepsilon'\cos\delta_m}{[(\mu'\cos\delta_e-\varepsilon'\cos\delta_m)^2+(\tan(\delta_m/2-\delta_e/2))^2(\mu'\cos\delta_e+\varepsilon'\cos\delta_m)^2]} \tag{S12}$$

Besides, based on the rule of $\sin^2\theta + \cos^2\theta = 1$, Eq. (5) and Eq. (6) can be rewritten as $\sinh^2(Kfd) = M$. Therefore, the

impedance matching degree can be assessed by the delta-function $\Delta = |\sinh^2(Kfd) - M|$.

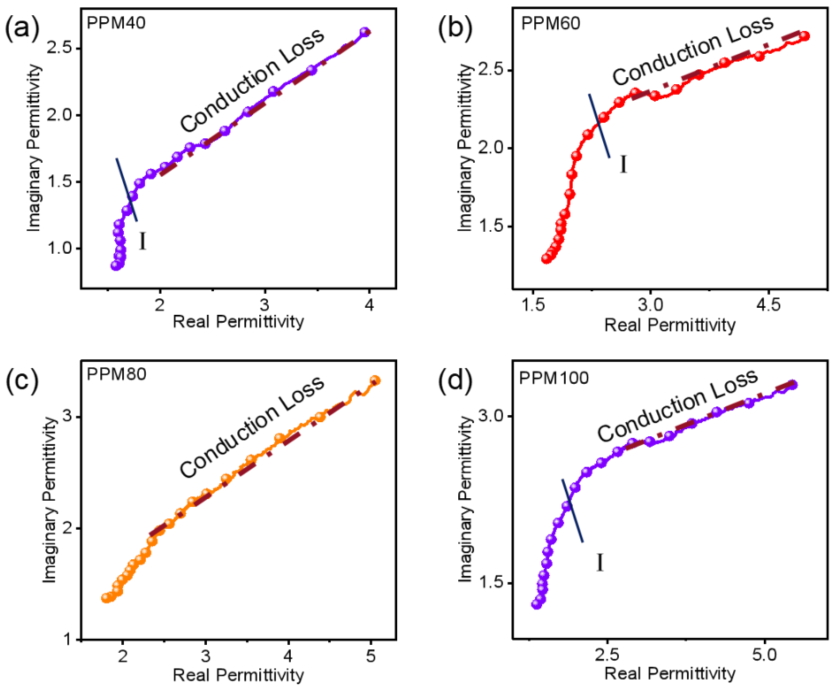

Figure S15. Cole-Cole plots of a) PPM40, b) PPM60, c) PPM80, and d) PPM100 samples.

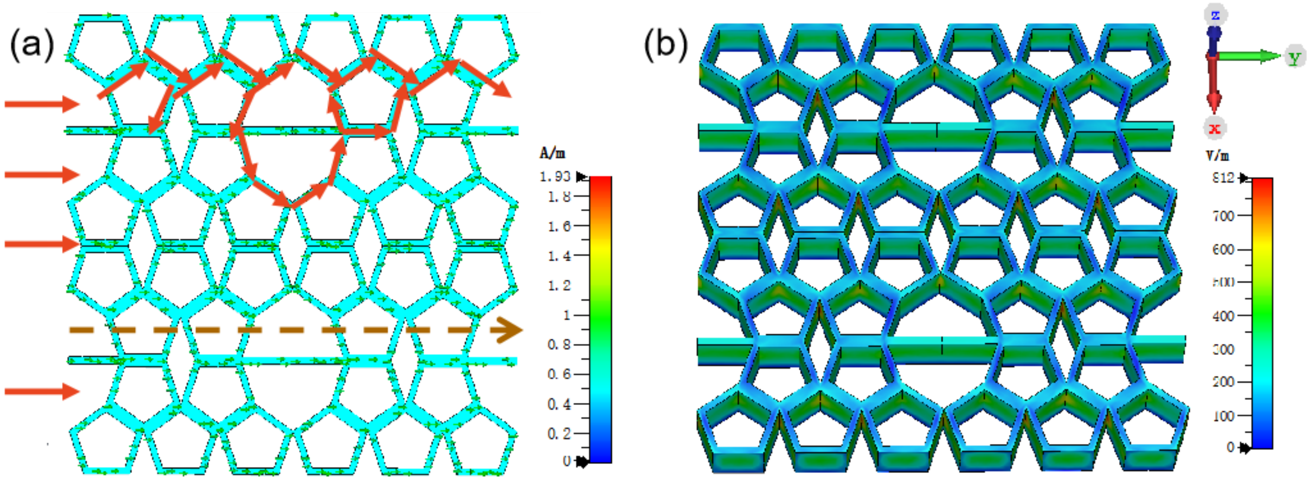

Figure S16. (a) Simulated electric field vector distribution on the upper surface of the simplified 3D network structure. (b) Electric field distribution of the porous foam sample at 10 GHz.

Radar equation and radar cross section (RCS) simulation theroy:

Radar, namely, wireless detection and stadiometry, is one of the most common applications of microwave technology. Fascinatingly, radar systems possess typical applications such as civilian use (airport surveillance, ocean navigation, weather radar, burglar alarm); detection utilization (air and sea navigation, spacecraft detection and tracking, missile guidance, weapon fuze); and scientific applications (astronomy, cartography and imaging, precise distance measurement, environmental remote sensing).[17,18] In accordance of different positions of receiving and transmitting radar waves, radar systems can be divided into monostatic (the same position) and bistatic (the different positions) radar systems.[19] As shown in Figure 6a and 6b, the abridged general view of the monostatic and bistatic radar systems exhibit that the antenna transmits signals and the target reflects or scatters after receiving these signals. Most radars are monostatic; however, the target is illuminated by separate transmitting antennas in some specific applications like the control of missile ignition. Therefore, taking the case of monostatic radar system as an example, following are the relevant equations: [20]

$$S_i = P_iG/4\pi R^2 \tag{S13}$$

Herein, S_i is the power density incident on target, P_i signifies radiant power, G represents the antenna gain, and R stands for the distance to the target. The target is assumed to be in the main beam direction of the antenna. There is no doubt that the target will scatter the incident power in all directions. The ratio of scattering power (P_s) to incident power density (S_i) in a given direction can be defined as the radar cross section of the target:

$$\sigma\ (\mathrm{m}^2) = P_s/S_i \tag{S14}$$

Thus, RCS can be intuitively understood as the characteristic of the target object itself, which has nothing to do with distance R. Besides, the dimension of RCS is area (m^2). Due to the dynamic range of radar cross section of target is very large, so it is often expressed by decibel square meters (dB m^2):

$$\sigma\ (\mathrm{dB\ m}^2) = 10\log\sigma(\mathrm{m}^2) \tag{S15}$$

Since the same target object will show different radar cross section characteristics for distinct radar frequency ranges, $k\alpha$ value has been introduced to manifest and analyze RCS values at different frequencies:

$$k\alpha = 2\pi\alpha/\lambda \qquad \text{(S16)}$$

Where $k = 2\pi/\lambda = 2\pi f/c$ is named as wave number, and α represents the feature size of the target. According to the different scattering characteristics of different wavelengths of electromagnetic wave irradiating the target object, the $k\alpha$ can be divided into three regions: (1) Rayleigh region ($k\alpha<0.5$); (2) resonance region ($0.5<k\alpha<10$); and (3) optical region ($k\alpha>10$).

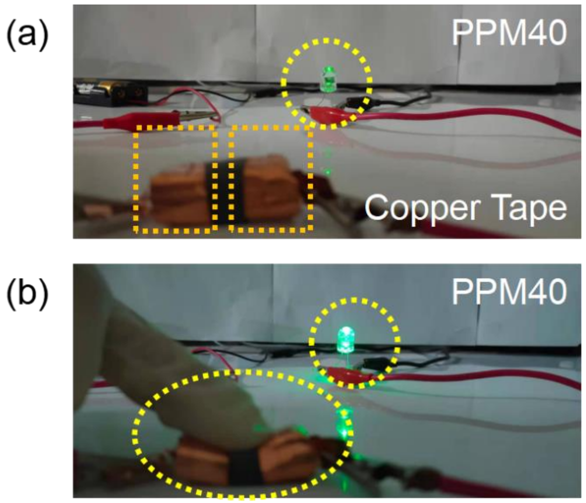

Figure S17. Digital photographs of the circuit with the conductive PPM40 foam show variation in the brightness of the LED a) without or b) with finger pressure.

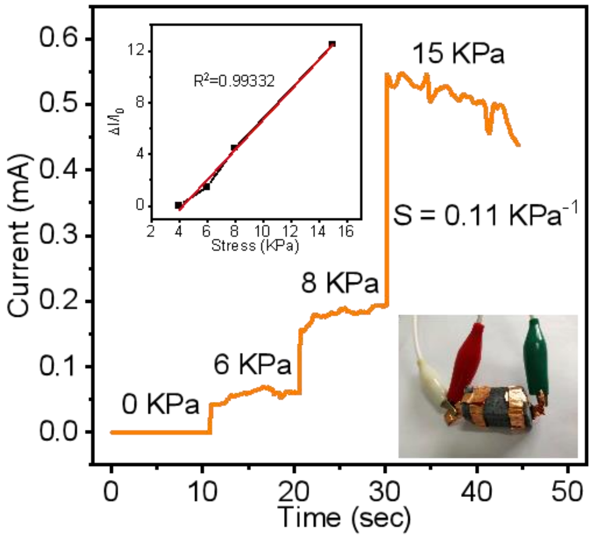

Figure S18. Real-time current signals recording of the circuit of PPM80 under different compressive pressure.

Tesla wireless transmission device and relevant mechanisms:

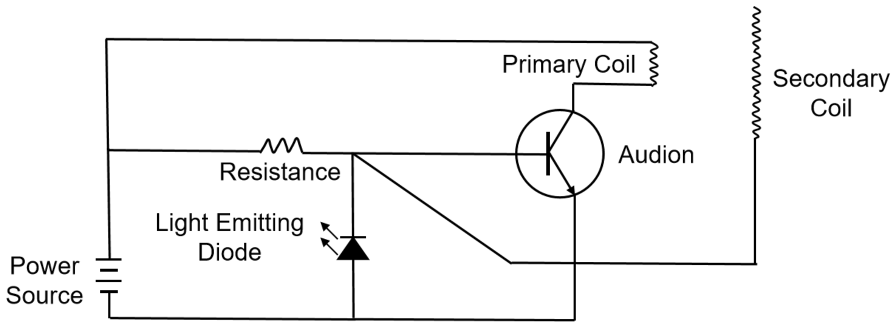

Figure S19. Schematic illustration of Tesla wireless transmission device.

Tesla wireless transmission, as one kind of energy transmission mode, can realize the energy transmission between power supply system and consumer via non-physical contact. [21] Essentially, Tesla coil is a step-up transformer, which can be found in **Figure S17**. From the perspective of mechanism, when the voltage transformer is boosted, the primary coil will pass through varying current, and the secondary coil will generate high voltage. Tesla wireless power transmission technology pertains to magnetic resonance technology, the corresponding mechanism is that the electrical energy sender and receiver coils constitute a combined magnetic resonance system. When the frequency of the oscillating magnetic field generated by the sending end is consistent with the natural frequency of the receiving end, the receiving end resonates, thus realizing the energy transmission. In a high voltage electric field, neon gas in a neon bubble gives of light by glow discharge. Thus, the neon bulb in **Figure 7**e can present a green light because of strong glow effect. However, if an electric conductor (electromagnetic shielding device) is placed between the power supply and demand objects, the resonance balance will be destroyed and then the electric energy transmission will be blocked. Namely, the neon bulb can not apply glow effect under this circumstance (**Figure 7**f).

REFERENCES

[1] C. Wang, X. Chen, B. Wang, M. Huang, B. Wang, Y. Jiang, R. Ruof, *ACS Nano* **2018**, *12*, 5816-5825.

[2] X. Li, X. Yin, C. Song, M. Han, H. Xu, W. Duan, L. Cheng, L. Zhang, *Adv. Funct. Mater.* **2018**, *28*, 1803938.

[3] S. Zhang, T. Tu, T. Li, Y. Cai, Z. Wang, Y. Zhou, D. Wang, L. Fang, X. Ye, Bo. Liang, *ACS Appl. Mater. Interfaces* **2022**, *14*, 23877-23887.

[4] G. Yang, S. Wang, H. Sun, X. Yao, C. Li, Y. Li, J. Jiang, *ACS Appl. Mater. Interfaces* **2021**, *13*, 57521-57531.

[5] J. Wang, X. Yue, X. Li, J. Dong, Q. Zhang, X. Zhao, *ACS Appl. Polym. Mater.* **2022**, *4*, 3205-3216.

[6] S. Tang, X. Zhang, J. Fan, B. Li, Z. Li, C. Wang, H. Li, P. Zhang, J. Zhou, *Applied Materials Today* **2022**, *26*, 101399.

[7] H. Tetik, K. Zhao, N. Shah, D. Lin, *J. Manuf Process* **2021**, *68*, 445-453.

[8] N. Yanagishima, S. Kanehashi, H. Saito, K. Ogino, T. Shimomura, *Polymer* **2020**, *206*, 122912.

[9] H. Huang, L. Xia, Y. Zhao, H. Zhang, T. Cong, J. Wang, N. Wen, S. Yang, Z. Fan, L. Pan, *Electrochim. Acta* **2020**, *364*, 137297.

[10] X. Zhao, W. Wang, Z. Wang, J. Wang, T. Huang, J. Dong, Q. Zhang, *Chem. Eng. J.* **2020**, *395*, 125115.

[11] F. Zhang, H. Hu, M. Islam, S. Peng, S. Wu, S. Lim, Y. Zhou, C. Wang, *Compos. Sci. Technol.* **2020**, *187*, 107959.

[12] T. Pham, S. Vadanan, S. Lim, *Cellulose* **2020**, *27*, 8075-8086.

[13] X. Qi, T. Miao, C. Chi, G. Zhang, C. Zhang, Y. Du, M. An, W. Ma, X. Zhang, *Nano Energy* **2020**, *77*, 105096.

[14] X. Jian, W. Tian, J. Li, L. Deng, Z. Zhou, L. Zhang, H. Lu, L. Yin, *ACS Appl. Mater. Interfaces* **2019**, *11*, 15869-15880.

[15] P. Miles, W. Westphal, A. Hippel, *Rev. Mod. Phys.* **1957**, *29*, 279-307.

[16] Z. Ma, C. Cao, A. Liu, J. Wang, *Chinese Phys. Lett.* **2012**, *29*, 038401.

[17] C. Li, Z. Peng, T. Huang, T. Fan, F. Wang, T. Horng, J. Munoz-Ferreras, R. Gomez-Garcia, L. Ran, J. Lin, *IEEE Trans. Microw. Theory* **2017**, *65*, 1692-1706.

[18] J. Hasch, E. Topak, R. Schnabel, T. Zwick, R. Weigel C. Waldschmidt, *IEEE Trans. Microw. Theory* **2012**, 60, 845-860.

[19] W. Chen, C. Balanis, C. Birtcher, *IEEE Trans. Antenn. Propag.* **2015**, *63*, 2636-2645.

[20] D. M. Pozar, Microwave Engineering, Fourth Edition, John Wiley & Sons, Inc. Hoboken, NJ, **2011**; pp 691-693.

[21] B. Zhou, M. Su, D. Yang, G. Han, Y. Feng, B. Wang, J. Ma, J. Ma, C. Liu, C. Shen, *ACS Appl. Mater. Interfaces* **2020**, *12*, 40859-40869.
